# Paeoniflorin and NAFLD: A Systematic Review and Meta‐Analysis of Animal Studies With Mechanistic Insights

**DOI:** 10.1002/fsn3.72192

**Published:** 2026-08-03

**Authors:** Dachuan Jin, Shunqin Jin, Tao Zhou, Guoping Sheng, Mingfei Yao, Peng Gao, Guangming Li, Chuan Qin

**Affiliations:** ^1^ Translational Medicine Research Center, Department of Liver Disease and Pharmacology Zhengzhou Sixth People's Hospital Zhengzhou China; ^2^ Department of Nuclear Medicine The First Medical Center, Chinese PLA General Hospital Beijing China; ^3^ Department of Geriatric Medicine & Laboratory of Gerontology and Anti‐Aging Research Qilu Hospital of Shandong University Jinan China; ^4^ Department of Infectious Diseases, Key Laboratory of Artificial Organs and Computational Medicine in Zhejiang Province Shulan (Hangzhou) Hospital, Shulan International Medical College, Zhejiang Shuren University Hangzhou China; ^5^ State Key Laboratory for Diagnosis and Treatment of Infectious Diseases, National Clinical Research Center for Infectious Diseases, Collaborative Innovation Center for Diagnosis and Treatment of Infectious Diseases The First Affiliated Hospital, College of Medicine, Zhejiang University Hangzhou China

**Keywords:** animal model, meta‐analysis, NAFLD, paeoniflorin, systematic review

## Abstract

Nonalcoholic fatty liver disease (NAFLD) is a major metabolic liver disorder with limited pharmacological options. Paeoniflorin (PF), a bioactive compound from *Paeonia lactiflora* , has shown hepatometabolic effects in experimental studies, but its overall efficacy in NAFLD models remains unclear. We searched PubMed, Embase, Web of Science, the Cochrane Library, CNKI, Wanfang, VIP, and CBM from inception to January 2026 for controlled animal studies evaluating PF in diet‐induced NAFLD models. Two reviewers independently performed study selection, data extraction, and risk‐of‐bias assessment using SYRCLE's tool. Weighted mean differences or standardized mean differences with 95% confidence intervals were pooled using random‐effects models. Ten studies were included, all using diet‐induced models. PF treatment was associated with improvements in lipid metabolism, liver injury, glucose homeostasis, inflammation, and oxidative stress, including reductions in total cholesterol, triglycerides, low‐density lipoprotein cholesterol, alanine aminotransferase, aspartate aminotransferase, body weight, fasting blood glucose, insulin resistance indices, tumor necrosis factor‐α, and malondialdehyde, together with increased superoxide dismutase activity. High‐density lipoprotein cholesterol showed no consistent improvement. Mechanistic findings suggested that PF may activate AMP‐activated protein kinase, inhibit sterol regulatory element‐binding protein‐1c/fatty acid synthase‐mediated lipogenesis, and modulate inflammatory and oxidative‐stress pathways. However, substantial heterogeneity and incomplete reporting of randomization, allocation concealment, and blinding limited confidence in the evidence. PF showed promising preclinical effects in NAFLD, but further well‐designed animal studies and clinical investigations are needed to clarify dose–response relationships, safety, and translational relevance.

## Graphical abstract

Evidence from 10 preclinical studies involving 181 rodents indicates that paeoniflorin may alleviate several features of nonalcoholic fatty liver disease, including dyslipidemia, impaired glucose metabolism, liver injury, inflammation, and oxidative stress. The available mechanistic data point mainly to AMPK activation and suppression of SREBP‐1c/FAS‐related lipogenesis. Further experimental and clinical studies are needed to determine whether these effects can be translated into therapeutic benefit.

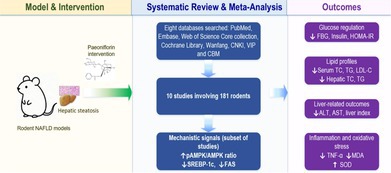

## 1 Introduction

Nonalcoholic fatty liver disease (NAFLD) is among the most common chronic liver diseases worldwide, affecting an estimated 25%–38% of the global population (Teng et al. 2023; Amini‐Salehi et al. 2024; Jin et al. 2023). Its increasing prevalence parallels the global rise in obesity, type 2 diabetes, and metabolic syndrome (Younossi and Henry 2022; Bisaccia et al. 2020). NAFLD encompasses a spectrum from simple steatosis to nonalcoholic steatohepatitis (NASH), which may progress to fibrosis, cirrhosis, and hepatocellular carcinoma (Pydyn et al. 2020; Dorairaj et al. 2021; Jin et al. 2025). Therapeutic options remain limited for many patients, and lifestyle modification remains the cornerstone of management despite well‐recognized challenges in long‐term adherence (Zhou et al. 2020; Stefan 2020; Thomas and Thomas 2025; Jin et al. 2022). Although disease‐modifying pharmacotherapy has begun to emerge for selected NASH populations, overall unmet need remains substantial (Petroni et al. 2024; Liu et al. 2025). In this review, we use the term NAFLD because the included preclinical animal studies were conducted and reported under the NAFLD/NASH framework, while MASLD is considered as updated terminology where relevant.

Paeoniflorin (PF), a defined bioactive constituent isolated from *Paeonia lactiflora* , has attracted interest in liver and metabolic disease research (Chen, Wu, and Wood 2013), with several recent preclinical studies examining its effects in NAFLD models (Shen et al. 2025; Zhang, Kong, et al. 2024; Li et al. 2018; Ma et al. 2020). These studies suggest that PF may improve key pathogenic domains relevant to NAFLD progression, including dysregulated lipid metabolism, oxidative stress, and inflammatory signaling (Shen et al. 2025; Gong et al. 2024; Lu et al. 2024). However, reported effects have not been fully consistent across animal studies, with discrepant findings for outcomes such as aminotransferases and body weight (Shen et al. 2025; Zhang, Kong, et al. 2024; Li et al. 2018). To date, evidence for PF in NAFLD remains predominantly preclinical, making it difficult to gauge the magnitude and robustness of its therapeutic signal and to identify study features that may explain variability (Ma et al. 2020). Therefore, we conducted a systematic review and meta‐analysis of animal studies to quantify the effects of PF on NAFLD‐related outcomes, to assess between‐study heterogeneity, and, where substantial heterogeneity was observed, to explore potential effect modifiers, including species, dosing regimen, treatment duration, and route of administration.

## 2 Materials and Methods

This preclinical meta‐analysis was registered in the PROSPERO database (Registration Number: CRD420251274050) and was conducted in accordance with the Preferred Reporting Items for Systematic Reviews and Meta‐Analysis (PRISMA) 2020 statement to ensure transparency and rigor in the review process (Page et al. 2021).

### 2.1 Search Strategy

A systematic search was performed in four English‐language databases (PubMed, Embase, Web of Science Core Collection, and the Cochrane Library) and four Chinese‐language databases (Wanfang, CNKI, VIP, and CBM) from inception to January 5, 2026. The search combined controlled vocabulary (e.g., MeSH/Emtree) and free‐text terms for “paeoniflorin” and “nonalcoholic fatty liver disease”, including the updated terminology “metabolic dysfunction‐associated steatotic liver disease” and related terms (e.g., NAFLD, NASH, hepatic steatosis, etc.). Reference lists of included studies and relevant reviews were manually screened to identify additional eligible studies. No language restrictions were applied. The full PubMed search strategy is presented in Table 1 as an example.

**TABLE 1:** Search strategy on PubMed.

#1	peoniflorin [Title/Abstract] OR paeoniflorin [Title/Abstract] OR peoniflorin sulfonate [Title/Abstract]
#2	Non‐alcoholic Fatty Liver Disease [MeSH Terms]
#3	Non‐alcoholic Fatty Liver Disease [Title/Abstract] OR Non alcoholic Fatty Liver Disease [Title/Abstract] OR Fatty Liver, Nonalcoholic [Title/Abstract] OR Fatty Livers, Nonalcoholic [Title/Abstract] OR Liver, Nonalcoholic Fatty [Title/Abstract] OR Livers, Nonalcoholic Fatty [Title/Abstract] OR Nonalcoholic Fatty Liver [Title/Abstract] OR Nonalcoholic Fatty Livers [Title/Abstract] OR NAFLD [Title/Abstract] OR Nonalcoholic Fatty Liver Disease [Title/Abstract] OR Nonalcoholic Steatohepatitis [Title/Abstract] OR Nonalcoholic Steatohepatitides [Title/Abstract] OR Steatohepatitides, Nonalcoholic [Title/Abstract] OR Steatohepatitis, Nonalcoholic [Title/Abstract] OR MAFLD [Title/Abstract] OR metabolic associated fatty liver disease [Title/Abstract] OR MASLD [Title/Abstract] OR metabolic dysfunction‐associated steatotic liver disease [Title/Abstract] OR metabolic dysfunction‐associated fatty liver disease [Title/Abstract] OR NAFLD[Title/Abstract] OR NASH [Title/Abstract] OR MASH [Title/Abstract] OR metabolic associated steatohepatitis [Title/Abstract] OR steatosis of liver [Title/Abstract] OR steatohepatitis nonalcoholic [Title/Abstract] OR metabolic associated steatohepatitis[Title/Abstract] OR liver steatosis [Title/Abstract]
#4	#2 OR #3
#5	#1 AND #4

### 2.2 Eligible Criteria

Inclusion criteria were based on the PICO framework: (1) population: in vivo controlled studies using rat or mouse models of NAFLD induced by validated dietary (e.g., high‐fat/fructose diets and methionine–choline‐deficient diet) or genetic (e.g., ob/ob and db/db) methods; (2) intervention: paeoniflorin administered as monotherapy (oral gavage or via diet/drinking water) for ≥ 4 weeks at any dose/regimen; (3) comparator: either a vehicle control (e.g., saline/water) or a no‐treatment control receiving the same diet without any gavage, or an identical diet/drinking water without paeoniflorin; and (4) outcomes: reporting at least one prespecified NAFLD outcome (e.g., liver enzymes, lipid/glucose/insulin‐resistance indices, oxidative‐stress or inflammatory markers, histology, or relevant signaling molecules) with extractable quantitative data. Only studies reporting data from independent experimental groups were eligible.

Exclusion criteria were as follows: (1) non‐NAFLD models; (2) additional experimentally induced comorbidities or unrelated disease models likely to confound effects; (3) paeoniflorin combined with other agents, unclear exposure (dose/duration not specified), or treatment < 4 weeks; (4) inappropriate or unclear control conditions; (5) non‐in vivo or non‐original studies (in vitro/ex vivo/in silico, human studies, reviews, case reports, conference abstracts, editorials); and (6) no relevant outcomes or insufficient data to calculate effect sizes.

### 2.3 Study Selection and Data Extraction

All retrieved records were imported into EndNote for management and de‐duplication. Two reviewers independently screened titles and abstracts to identify potentially eligible studies, followed by full‐text assessment against the predefined criteria. Any disagreements were resolved through discussion; if consensus could not be reached, a third senior reviewer adjudicated.

Data were extracted independently by two reviewers using a standardized extraction form, including: first author, publication year, country, study design; animal characteristics (species/strain, sex, age/weight, and sample size); NAFLD induction method and criteria for successful modeling; intervention details (dose, route, frequency, and duration); comparator details; and outcome data (means and dispersion measures). When multiple time points were reported, data from the latest time point (end of the intervention period) were preferentially extracted. When multiple paeoniflorin dose arms were reported in the same study, we a priori extracted data from the highest‐dose arm to avoid unit‐of‐analysis issues arising from shared control groups and to maintain statistical independence, consistent with previous preclinical meta‐analyses in similar settings (Jiang et al. 2024). When data were presented graphically, values were extracted using digital ruler software (e.g., GetData Graph Digitizer).

### 2.4 Quality Assessment

SYRCLE's Risk of Bias tool was applied to evaluate methodological quality across 10 bias domains: sequence generation, baseline characteristics, allocation concealment, random housing, blinding of both caregivers and investigators, random outcome assessment, blinding of outcome assessors, incomplete outcome data, selective reporting of outcomes, and other sources of bias (Hooijmans et al. 2014).

For each domain, studies were classified as having either a “low,” “high,” or “unclear” risk of bias based on the methodological details provided. Any disagreements between the two reviewers were resolved through discussion, and in cases where consensus could not be reached, a senior reviewer was consulted for further clarification.

### 2.5 Statistical Analysis

Meta‐analyses were conducted only when at least three independent datasets (typically from three or more studies) were available for a given outcome to avoid unstable variance estimates and low statistical power (Herbison et al. 2011). Effect sizes were summarized as weighted mean differences (WMDs) for outcomes measured on the same scale or convertible to a common unit (with unit harmonization performed prior to pooling). For outcomes not directly comparable across studies—particularly assay‐dependent measurements (e.g., ALT, AST, insulin, HOMA‐IR, inflammatory and oxidative stress markers, and signaling protein/mRNA expression)—standardized mean differences (SMDs) were used. Outcomes were classified a priori into physiological‐quantity versus assay‐dependent categories, and the choice of WMD or SMD followed this prespecified classification rather than post hoc unit considerations.

A random‐effects model was prespecified for all analyses due to the inherent methodological and biological diversity of animal studies. Effect sizes were calculated using the inverse‐variance method. The degree of heterogeneity was assessed using the *I* ^2^ statistic, where values less than 50% were considered indicative of low heterogeneity, and values greater than or equal to 50% indicated high heterogeneity.

For outcomes with high heterogeneity (*I* ^2^ ≥ 50%), meta‐regression analysis was planned for those with 10 or more studies, as such analyses are generally considered more reliable when a sufficient number of studies are available (Chandler et al. 2019). For outcomes with fewer than 10 but at least three studies, prespecified subgroup analyses were conducted to explore potential sources of heterogeneity according to study‐level characteristics, including animal species, paeoniflorin dose (low dose: < 100 mg/kg/day, high dose: ≥ 100 mg/kg/day), treatment duration (short‐term: ≤ 8 weeks, long‐term: > 8 weeks), and route of administration (oral gavage vs. mixed with drinking water) (Sun et al. 2025). When a study administered multiple paeoniflorin doses, only the highest‐dose arm was extracted for meta‐analysis for a given outcome to avoid unit‐of‐analysis issues arising from shared control groups, and the study was assigned to the corresponding dose subgroup based on the highest dose administered between studies using higher versus lower doses, rather than within‐study dose–response comparisons.

To examine whether selection of the highest‐dose arm influenced the pooled estimates, we performed post hoc sensitivity analyses using the lowest available PF dose arm instead of the highest‐dose arm for outcomes in which at least one included study reported multiple eligible PF dose arms. These analyses were conducted using the same effect‐size metric and random‐effects model as the primary analyses. The purpose of these sensitivity analyses was not to estimate a formal within‐study dose–response relationship, but to evaluate whether the direction and robustness of the pooled findings were influenced by preferential extraction of the highest‐dose arm.

Publication bias was assessed using funnel plots and Egger's regression asymmetry test and Begg's rank correlation test, but these analyses were only performed for outcomes with 10 or more studies, in line with the Cochrane Handbook for Systematic Reviews of Interventions (version 6.2), which suggests that such tests are unreliable with fewer studies (Chandler et al. 2019). For outcomes with at least five studies, leave‐one‐out sensitivity analyses and Galbraith plots were performed to assess the robustness of the pooled estimates and to identify studies that may contribute disproportionately to between‐study heterogeneity. In the leave‐one‐out analyses, the meta‐analysis was repeated after sequentially omitting one study at a time. Galbraith plots were used as influence diagnostics and were not used as a basis for excluding studies unless clear data extraction or eligibility errors were identified (Chandler et al. 2019; Hu et al. 2022). All statistical analyses were performed using STATA 15.1 (StataCorp, College Station, TX). A two‐tailed *p* < 0.05 was considered statistically significant.

## 3 Results

### 3.1 Study Selection

The study selection process is summarized in the PRISMA 2020 flow diagram (Figure S1). Our initial search identified a total of 151 records from multiple databases, including PubMed, Embase, Web of Science, Cochrane Library, CNKI, Wanfang, VIP, and CBM. After removing duplicates (*n* = 39), 112 records were screened based on titles and abstracts. A further 71 studies were excluded, and 41 reports were retrieved for full‐text assessment. Following full‐text review, 31 studies were excluded for reasons including being conference abstracts (*n* = 3), in vitro studies (*n* = 9), review articles (*n* = 7), irrelevant articles (*n* = 5), and patents or protocols (*n* = 7). Ultimately, 10 studies were included in the qualitative synthesis and meta‐analysis (Shen et al. 2025; Zhang, Kong, et al. 2024; Li et al. 2018; Chen, Zhang, et al. 2013; Guo et al. 2025; Liu et al. 2022; Ma et al. 2016; Ma et al. 2017; Zhang et al. 2015; Zhao et al. 2025). In addition, three studies were identified through other sources such as websites and citation searching, but all were excluded as they did not meet the inclusion criteria.

### 3.2 Characteristics of Included Studies

The characteristics of the included studies are summarized in Table 2. All studies focused on animal models of NAFLD, with a majority using Sprague–Dawley rats (*n* = 6) (Zhang, Kong, et al. 2024; Li et al. 2018; Chen, Zhang, et al. 2013; Liu et al. 2022; Ma et al. 2016; Ma et al. 2017) and the remainder using C57BL/6J mice (*n* = 4) (Shen et al. 2025; Guo et al. 2025; Zhang et al. 2015; Zhao et al. 2025). The animals were induced with NAFLD using high‐fat diets (HFD, *n* = 9) (Shen et al. 2025; Zhang, Kong, et al. 2024; Chen, Zhang, et al. 2013; Guo et al. 2025; Liu et al. 2022; Ma et al. 2016; Ma et al. 2017; Zhang et al. 2015; Zhao et al. 2025) or fructose (*n* = 1) (Li et al. 2018). The number of animals per group ranged from 6 to 11.

**TABLE 2:** Characteristics of included studies.

Author	Species (Strain)	Model (Inducer)	n (E/C)	Dose of PF (mg/kg/d)	ROA	T (w)	Outcome measures
Chen2013	Rat (SD)	NAFLD (HFD)	10/11	200	Gavage	4	ALT, AST, FBG, INS, pAMPK/AMPK ratio, SREBP‐1c, FAS
Ma2017	Rat (SD)	NAFLD (HFD)	8/8	20	Gavage	4	BW, liver index, ALT, AST, hepatic TG/TC, serumTG/TC/LDL‐C/HDL‐C, FBG, INS, HOMA‐IR, SOD, MDA
Guo2025	Mice (C57B//6J)	NAFLD (HFD)	10/10	100	Gavage	8	liver index, ALT, AST, hepatic TG/TC, serum TG/TC, FBG, INS, HOMA‐IR, SOD, MDA
Li2018	Rat (SD)	NAFLD (Fructose)	8/8	40	Gavage	8	BW, ALT, AST, hepatic TG, serum TG/TC/LDL‐C/HDL‐C, pAMPK/AMPK ratio, SREBP‐1c, FAS
Ma2016	Rat (SD)	NAFLD (HFD)	10/10	100	Gavage	4	BW, liver index, ALT, AST, hepatic TG/TC, serum TG/TC/LDL‐C/HDL‐C, TNF‐α
Zhang2015	Mice (C57BL/6J)	NAFLD (HFD)	10/10	34	Dietary	24	BW, liver index, ALT, AST, hepatic TG/TC, serum TG/TC/LDL‐C/HDL‐C, h‐TC, FBG, INS, HOMA‐IR, SREBP‐1c, FAS
Zhang2024	Rat (SD)	NAFLD (HFD)	8/8	50	Gavage	4	ALT, AST, serum TG/TC, TNF‐α
Zhao2025	Mice (C57BL/6J)	NAFLD (HFD)	10/10	60	Gavage	4	Serum TG/TC/LDL‐C/HDL‐C, TNF‐α
Liu2022	Rat (SD)	NAFLD (HFD)	10/10	20	Gavage	4	BW, liver index, ALT, AST, hepatic TG/TC, serum TG/TC/LDL‐C/HDL‐C, TNF‐α, SOD, MDA, pAMPK/AMPK ratio
Shen2025	Mice (C57BL/6J)	NAFLD (HFD)	6/6	50	Gavage	8	BW, liver index, ALT, AST

Abbreviations: ALT, alanine aminotransferase; AST, aspartate aminotransferase; BW, body weight; FBG, fasting blood glucose; HDL‐C, high‐density lipoprotein cholesterol; INS, insulin; LDL‐C, low‐density lipoprotein cholesterol; MDA, malondialdehyde; SOD, superoxide dismutase; TC, total cholesterol; TG, triglycerides; TNF, tumor necrosis factor.

The administered dose of Paeoniflorin (PF) varied from 20 mg/kg/day to 200 mg/kg/day. The majority of the studies (*n* = 9) (Shen et al. 2025; Zhang, Kong, et al. 2024; Li et al. 2018; Chen, Zhang, et al. 2013; Guo et al. 2025; Liu et al. 2022; Ma et al. 2016; Ma et al. 2017; Zhao et al. 2025) used oral gavage as the route of administration, while one study (Zhang et al. 2015) used dietary supplementation. Treatment durations ranged from 4 to 24 weeks, with most studies (*n* = 9) (Shen et al. 2025; Zhang, Kong, et al. 2024; Li et al. 2018; Chen, Zhang, et al. 2013; Guo et al. 2025; Liu et al. 2022; Ma et al. 2016; Ma et al. 2017; Zhao et al. 2025) using a treatment period of 4–8 weeks.

Outcome measures varied widely, with key parameters including liver function markers (such as ALT and AST), oxidative stress markers (SOD and MDA), and inflammation markers (TNF‐α). Other common outcomes included anthropometric markers (BW and liver index), lipid profiles (serum TG, TC, LDL‐C, and HDL‐C, as well as hepatic TC and TG), and glucose metabolism markers (FBG, INS, and HOMA‐IR). Additionally, signaling molecules related to liver metabolism, such as NF‐κB, pAMPK ratio, and FAS, were assessed in several studies.

### 3.3 Risk of Bias and Quality of the Study

The quality assessment of the 10 included studies is presented in Figures S2 and S3. Moderate risk of bias was identified in several areas. Specifically, nine studies were assessed as having unclear risk in random sequence generation (selection bias), random outcome assessment (detection bias), and blinding (detection bias), contributing to a moderate risk of bias. Additionally, all 10 studies were judged to have unclear risk of bias in blinding (performance bias) and allocation concealment (selection bias), leading to a moderate risk classification. Moreover, four studies were found to have unclear risk in baseline characteristics (selection bias) and random housing (performance bias), resulting in a moderate risk. All 10 studies were rated as having low risk in incomplete outcome data (attrition bias), selective reporting (reporting bias), and other bias. Because inadequate reporting of randomization and blinding may exaggerate treatment effects in animal experiments, the pooled estimates may represent optimistic estimates of efficacy.

### 3.4 Meta‐Analysis Results

This meta‐analysis assessed the effects of Paeoniflorin (PF) on various physiological and biochemical outcomes in animal models of NAFLD, with a focus on lipid metabolism, liver function, glucose metabolism, anthropometrics, inflammation, oxidative stress markers, and signaling molecules. All analyses were conducted using a random‐effects model due to the high heterogeneity observed across the studies. The heterogeneity for each outcome was assessed using the *I* ^2^ statistic, with a significance threshold of *p* < 0.05.

#### 3.4.1 Lipid Metabolism

**FIGURE 1:**
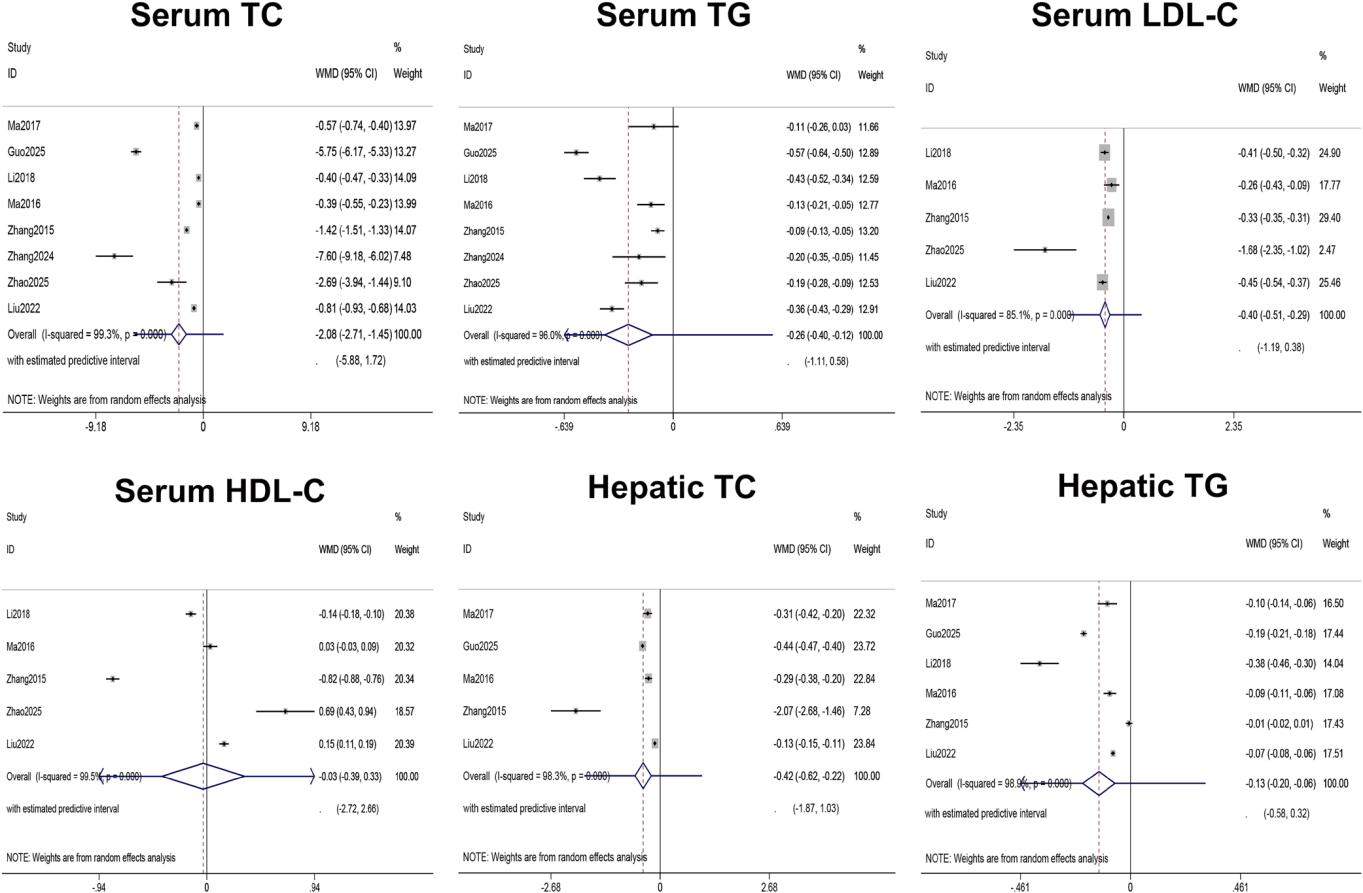
Forest plots of the effects of PF on lipid metabolism outcomes in animal models of NAFLD. HDL‐C, high‐density lipoprotein cholesterol; LDL‐C, low‐density lipoprotein cholesterol; NAFLD, nonalcoholic fatty liver disease; PF, paeoniflorin; TC, total cholesterol; TG, triglycerides.

PF was associated with improvements in several lipid metabolism markers, including both serum and hepatic lipid profiles (Figure 1). The analysis of serum total cholesterol (TC) from eight studies (Zhang, Kong, et al. 2024; Li et al. 2018; Guo et al. 2025; Liu et al. 2022; Ma et al. 2016; Ma et al. 2017; Zhang et al. 2015; Zhao et al. 2025), revealed a pooled WMD of −2.08 mmol/L (95% CI: −2.71 to −1.45; *p* < 0.001), with high heterogeneity (*I* ^2^ = 99.3%, *p* < 0.001). This suggests that PF was associated with lower serum total cholesterol levels in the included animal models. Similarly, serum triglycerides (TG) showed a reduction with a pooled WMD of −0.26 mmol/L (95% CI: −0.40 to −0.12; *p* < 0.001) across eight studies (Zhang, Kong, et al. 2024; Li et al. 2018; Guo et al. 2025; Liu et al. 2022; Ma et al. 2016; Ma et al. 2017; Zhang et al. 2015; Zhao et al. 2025), with significant heterogeneity (*I* ^2^ = 96.0%, *p* < 0.001), supporting an association between PF treatment and lower triglyceride levels in NAFLD models. For serum LDL‐C, a pooled WMD of −0.40 mmol/L (95% CI: −0.51 to −0.29; *p* < 0.001) was found across five studies (Li et al. 2018; Liu et al. 2022; Ma et al. 2016; Zhang et al. 2015; Zhao et al. 2025), with high heterogeneity (*I* ^2^ = 85.1%, *p* < 0.001), indicating a directionally consistent reduction in LDL cholesterol despite substantial between‐study heterogeneity. However, the effect on serum HDL‐C was inconsistent across studies, with no statistically significant effect (WMD = −0.03 mmol/L, 95% CI: −0.39 to 0.33; *p* = 0.865) across five studies (Li et al. 2018; Liu et al. 2022; Ma et al. 2016; Zhang et al. 2015; Zhao et al. 2025), and significant heterogeneity (*I* ^2^ = 99.5%, *p* < 0.001). In hepatic lipid profiles, PF was also associated with lower hepatic total cholesterol and hepatic triglycerides. The pooled WMD for hepatic total cholesterol was −0.42 mmol/L (95% CI: −0.62 to −0.22; *p* < 0.001) from five studies (Guo et al. 2025; Liu et al. 2022; Ma et al. 2016; Ma et al. 2017; Zhang et al. 2015), with high heterogeneity (*I* ^2^ = 98.3%, *p* < 0.001). For hepatic triglycerides, six studies (Li et al. 2018; Guo et al. 2025; Liu et al. 2022; Ma et al. 2016; Ma et al. 2017; Zhang et al. 2015) were pooled, yielding a WMD of −0.13 mmol/L (95% CI: −0.20 to −0.06; *p* < 0.001) and high heterogeneity (*I* ^2^ = 98.9%, *p* < 0.001), further supporting a potential association between PF treatment and reduced hepatic lipid accumulation.

#### 3.4.2 Liver Enzymes

**FIGURE 2:**
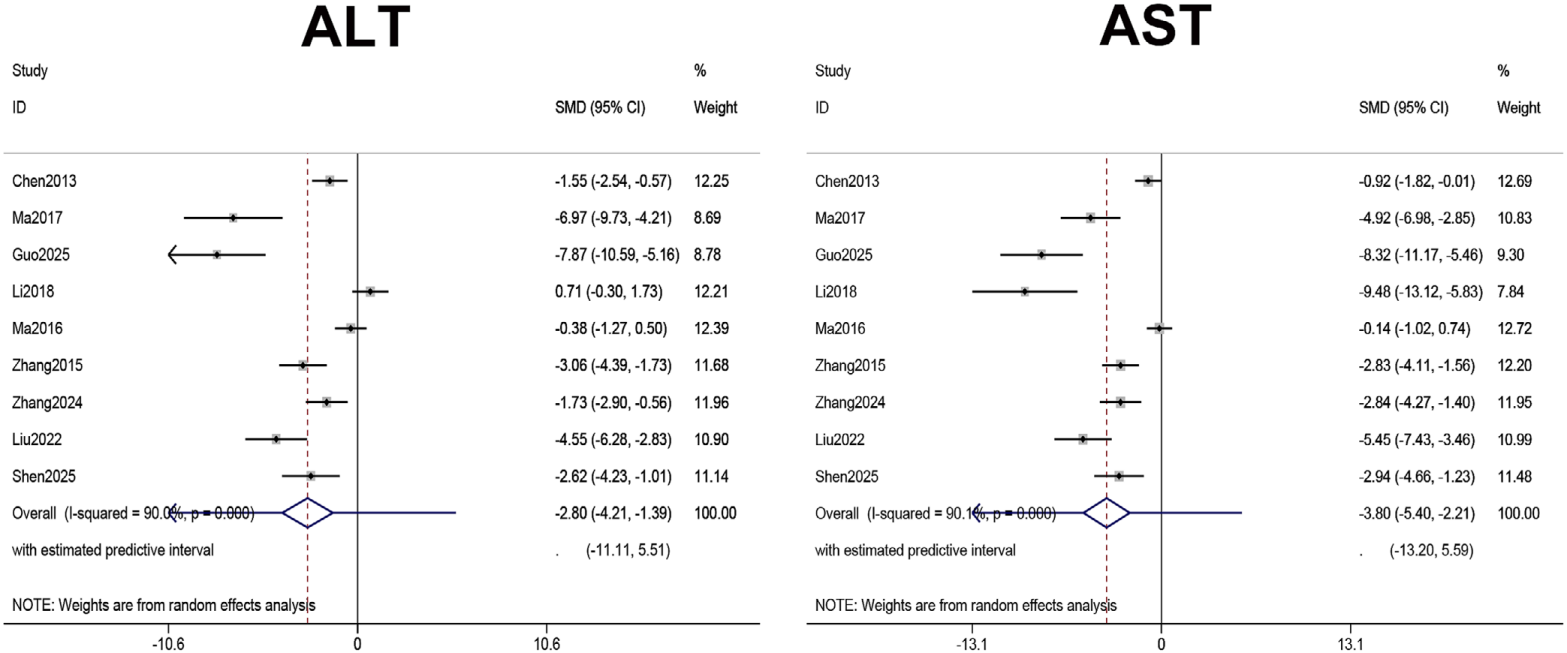
Forest plots of the effects of PF on liver enzyme outcomes in animal models of NAFLD. ALT, alanine aminotransferase; AST, aspartate aminotransferase; NAFLD, nonalcoholic fatty liver disease; PF, paeoniflorin.

PF was associated with lower liver enzyme levels, including alanine aminotransferase (ALT) and aspartate aminotransferase (AST), across the included animal studies (Figure 2). For ALT, nine studies (Shen et al. 2025; Zhang, Kong, et al. 2024; Li et al. 2018; Chen, Zhang, et al. 2013; Guo et al. 2025; Liu et al. 2022; Ma et al. 2016; Ma et al. 2017; Zhang et al. 2015) were pooled, yielding a SMD of −2.80 (95% CI: −4.21 to −1.39; *p* < 0.001) with high heterogeneity (*I* ^2^ = 90.0%, *p* < 0.001). Similarly, AST was pooled from nine studies (Shen et al. 2025; Zhang, Kong, et al. 2024; Li et al. 2018; Chen, Zhang, et al. 2013; Guo et al. 2025; Liu et al. 2022; Ma et al. 2016; Ma et al. 2017; Zhang et al. 2015), with an SMD of −3.80 (95% CI: −5.40 to −2.21; *p* < 0.001) and high heterogeneity (*I* ^2^ = 90.1%, *p* < 0.001). Despite the consistent direction of effect for both ALT and AST, the magnitude of these pooled estimates should be interpreted cautiously because of the substantial between‐study heterogeneity.

#### 3.4.3 Anthropometric Indices

**FIGURE 3:**
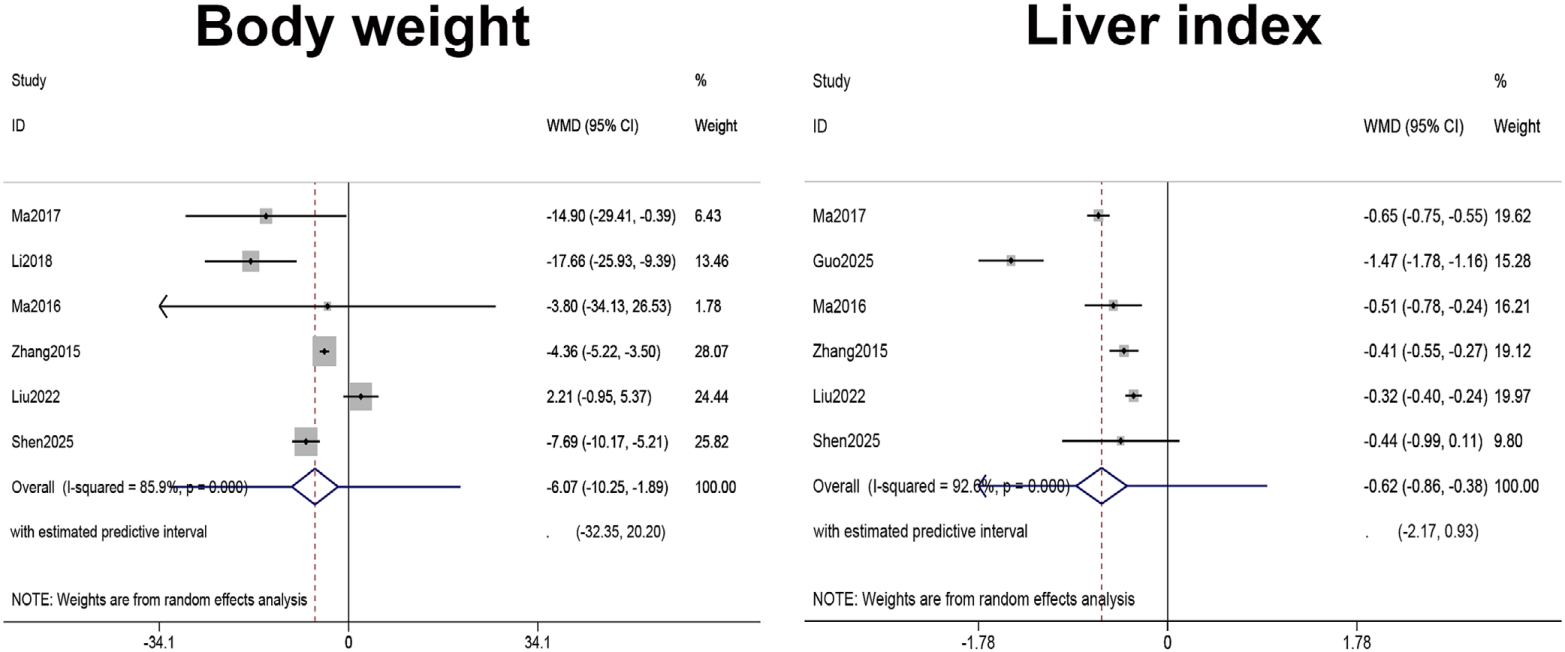
Forest plots of the effects of PF on anthropometric outcomes in animal models of NAFLD. BW, body weight; NAFLD, nonalcoholic fatty liver disease; PF, paeoniflorin.

The effects of PF on body weight (BW) and liver index are shown in Figure 3. For BW, the pooled WMD from six studies (Shen et al. 2025; Li et al. 2018; Liu et al. 2022; Ma et al. 2016; Ma et al. 2017; Zhang et al. 2015) was −6.07 g (95% CI: −10.25 to −1.89; *p* = 0.004), although heterogeneity was high (*I* ^2^ = 85.9%, *p* < 0.001). Liver index, which reflects liver size, was also significantly reduced by PF (WMD: −0.62%, 95% CI: −0.86 to −0.38; *p* < 0.001) across six studies (*N* = 6) (Shen et al. 2025; Guo et al. 2025; Liu et al. 2022; Ma et al. 2016; Ma et al. 2017; Zhang et al. 2015), with high heterogeneity (*I* ^2^ = 92.6%, *p* < 0.001). These findings suggest that PF may improve both body weight and liver enlargement in NAFLD animal models, although the magnitude of these effects should be interpreted cautiously because of substantial heterogeneity.

#### 3.4.4 Glucose Metabolism

**FIGURE 4:**
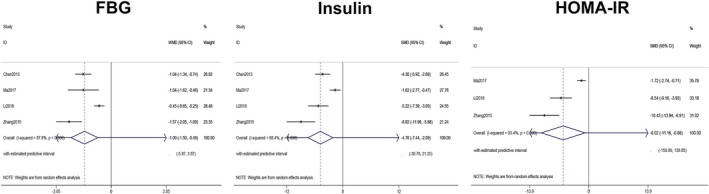
Forest plots of the effects of PF on glucose metabolism outcomes in animal models of NAFLD. FBG, fasting blood glucose; HOMA‐IR, homeostatic model assessment of insulin resistance; INS, insulin; NAFLD, nonalcoholic fatty liver disease; PF, paeoniflorin.

PF was associated with favorable changes in markers of glucose metabolism (Figure 4). For fasting blood glucose (FBG), the pooled WMD was −1.00 mmol/L (95% CI: −1.50 to −0.49; *p* < 0.001) across four studies (Li et al. 2018; Chen, Zhang, et al. 2013; Ma et al. 2017; Zhang et al. 2015), with high heterogeneity observed (I^2^ = 87.8%, *p* < 0.001). Serum insulin levels were also significantly reduced (SMD: −4.76, 95% CI: −7.44 to −2.09; *p* < 0.001) across four studies (Li et al. 2018; Chen, Zhang, et al. 2013; Ma et al. 2017; Zhang et al. 2015), with high heterogeneity (*I* ^2^ = 88.4%, *p* < 0.001). Similarly, HOMA‐IR, a marker of insulin resistance, showed a significant reduction (SMD: −6.02, 95% CI: −11.16 to −0.88; *p* = 0.022) across three studies (Li et al. 2018; Ma et al. 2017; Zhang et al. 2015), with very high heterogeneity (*I* ^2^ = 93.4%, *p* < 0.001).

#### 3.4.5 Inflammation and Oxidative Stress Markers

**FIGURE 5:**
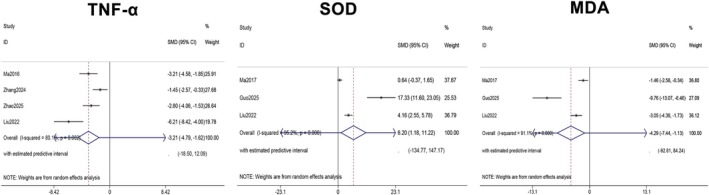
Forest plots of the effects of PF on inflammatory and oxidative stress outcomes in animal models of NAFLD. MDA, malondialdehyde; NAFLD, nonalcoholic fatty liver disease; PF, paeoniflorin; SOD, superoxide dismutase; TNF, tumor necrosis factor.

PF was associated with favorable changes in inflammatory and oxidative stress markers in NAFLD models (Figure 5). Regarding inflammation, PF significantly reduced the pro‐inflammatory cytokine TNF‐α, with a pooled SMD of −3.21 (95% CI: −4.79 to −1.62; *p* < 0.001) across four studies (Zhang, Kong, et al. 2024; Liu et al. 2022; Ma et al. 2016; Zhao et al. 2025), although substantial heterogeneity was observed (*I* ^2^ = 80.1%, *p* = 0.002). In terms of oxidative stress, PF significantly increased antioxidant capacity, as reflected by an elevated SOD level (SMD = 6.20, 95% CI: 1.18–11.22; *p* = 0.015) across three studies (Guo et al. 2025; Liu et al. 2022; Ma et al. 2017), and concurrently reduced lipid peroxidation, as reflected by decreased MDA levels (SMD = −4.29, 95% CI: −7.44 to −1.13; *p* = 0.008) across the same three studies (Guo et al. 2025; Liu et al. 2022; Ma et al. 2017). Collectively, these findings suggest that PF may be associated with reduced inflammatory and oxidative stress responses in NAFLD animal models.

#### 3.4.6 Signaling Molecules

**FIGURE 6:**
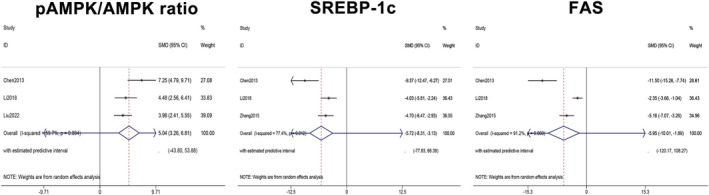
Forest plots of the effects of PF on signaling molecule outcomes in animal models of NAFLD. NAFLD, nonalcoholic fatty liver disease; pAMPK, pPhosphorylated AMP‐activated protein kinase; PF, paeoniflorin.

PF was also associated with changes in signaling molecules involved in liver metabolism (Figure 6). For pAMPK/AMPK ratio, the pooled SMD across three studies (Li et al. 2018; Chen, Zhang, et al. 2013; Liu et al. 2022) was 5.04 (95% CI: 3.26–6.81; *p* < 0.001), with high heterogeneity (*I* ^2^ = 59.7%, *p* = 0.084), suggesting that PF may be associated with activation of AMPK signaling. Similarly, SREBP‐1c, a key regulator of lipogenesis, was significantly reduced by PF (SMD: −5.72, 95% CI: −8.31 to −3.13; *p* < 0.001) in three studies (Li et al. 2018; Chen, Zhang, et al. 2013; Zhang et al. 2015), with substantial heterogeneity (*I* ^2^ = 77.4%, *p* = 0.012). Lastly, FAS levels were also reduced by PF, with a pooled SMD of −5.95 (95% CI: −10.01 to −1.89; *p* = 0.004) from three studies (Li et al. 2018; Chen, Zhang, et al. 2013; Zhang et al. 2015), showing high heterogeneity (*I* ^2^ = 91.2%, *p* < 0.001).

### 3.5 Subgroup Analysis

To investigate the sources of heterogeneity, subgroup analyses were conducted based on species, dosage, treatment duration, and route of administration (Tables 3, 4, 5, 6). Because not all outcomes were eligible for every subgroup factor (e.g., some outcomes had only one dose category across studies), the number of outcomes included in each subgroup analysis varied by factor. These analyses revealed that these factors contributed to variability in outcomes. For species, stratification into rats and mice reduced heterogeneity for 14 of the 15 outcomes, indicating that species differences were a significant source of variability (Table 3). Regarding dosage, a dose‐dependent effect was observed, with higher doses generally yielding more pronounced improvements in lipid metabolism (e.g., serum TC/TG and hepatic TC/TG). Among 18 outcomes, 12 showed reduced heterogeneity when stratified by low (< 100 mg/kg/day) and high (≥ 100 mg/kg/day) doses, highlighting the importance of dosage in influencing PF's efficacy (Table 4). In terms of treatment duration, subgroup analysis of 15 outcomes revealed that stratification by duration led to reduced heterogeneity in 10 out of the 15 outcomes. Longer treatment durations (> 8 weeks) were associated with more significant effects in some lipid metabolism markers (serum TC, LDL‐C, and HDL‐C) and ALT (Table 5). The route of administration also played a role, with 10 out of 15 outcomes showing reduced heterogeneity when categorized by oral gavage or mixed drinking water (Table 6).

**TABLE 3:** Subgroup analysis by species.

Category	Indicators	Species	No. of Exp.	Heterogeneity (*p value*)	SMD/WMD (95% CI)	*p*
Lipid metabolism	Serum TC	Rat	6	95.9% (< 0.001)	−0.99 (−1.36, −0.62)	< 0.001
		Mice	2	99.7% (< 0.001)	−3.58 (−7.82, 0.66)	0.098
	Serum TG	Rat	6	86.5% (< 0.001)	−0.24 (−0.35, −0.13)	< 0.001
		Mice	2	99.2% (< 0.001)	−0.33 (−0.79, 0.14)	0.170
	LDL‐C	Rat	4	83.4% (< 0.001)	−0.45 (−0.63, −0.28)	< 0.001
		Mice	1	NA	−0.33 (−0.35, −0.31)	< 0.001
	HDL‐C	Rat	4	97.3% (< 0.001)	0.14 (−0.05, 0.32)	0.152
		Mice	1	NA	−0.82 (−0.88, −0.76)	< 0.001
	Hepatic TC	Rat	3	90.6% (< 0.001)	−0.24 (−0.37, −0.10)	0.001
		Mice	2	96.4% (< 0.001)	−1.22 (−2.82, 0.38)	0.134
	Hepatic TG	Rat	4	94.7% (< 0.001)	−0.14 (−0.21, −0.08)	< 0.001
		Mice	2	99.7% (< 0.001)	−0.10 (−0.29, 0.09)	0.290
Glucose metabolism	FBG	Rat	3	83.4% (0.002)	−0.81 (−1.27, −0.35)	0.001
		Mice	1	NA	−1.57 (−2.05, −1.09)	< 0.001
	INS	Rat	3	83.4% (0.002)	−3.59 (−5.87, −1.31)	0.002
		Mice	1	NA	−8.92 (−11.96, −5.88)	< 0.001
	HOMA‐IR	Rat	2	91.2% (0.001)	−3.98 (−8.69, 0.74)	0.098
		Mice	1	NA	−10.43 (−13.94, −6.91)	< 0.001
Liver enzymes	ALT	Rat	7	88.5% (< 0.001)	−2.15 (−3.56, −0.74)	0.003
		Mice	2	90.0% (0.002)	−5.32 (−10.02, −0.61)	0.027
	AST	Rat	7	90.0% (< 0.001)	−3.40 (−5.16, −1.65)	< 0.001
		Mice	2	91.5% (0.001)	−5.42 (−10.79, −0.05)	0.048
Anthropometrics	BW	Rat	4	87% (< 0.001)	−8.68 (−22.46, 5.10)	0.217
		Mice	2	83.8% (0.013)	−5.81 (−9.05, −2.58)	< 0.001
	Liver index	Rat	4	87.9% (< 0.001)	−0.48 (−0.71, −0.25)	< 0.001
		Mice	2	97.4% (< 0.001)	−0.93 (−1.97, 0.11)	0.079
Oxidative stress	SOD	Rat	2	92.4% (< 0.001)	2.34 (−1.11, 5.79)	0.183
		Mice	1	NA	17.33 (11.60, 23.05)	< 0.001
	MDA	Rat	2	68.9% (0.073)	−2.22 (−3.77, −0.66)	0.005
		Mice	1	NA	−9.76 (−13.07, −6.46)	< 0.001

Abbreviations: ALT, alanine aminotransferase; AST, aspartate aminotransferase; BW, body weight; FBG, fasting blood glucose; HDL‐C, high‐density lipoprotein cholesterol; INS, insulin; LDL‐C, low‐density lipoprotein cholesterol; MDA, malondialdehyde; SOD, superoxide dismutase; TC, total cholesterol; TG, triglycerides.

**TABLE 4:** Subgroup analysis by dosage.

Category	Indicators	Dosage	Number of experiments	Heterogeneity (*p* value)	SMD/WMD (95% CI)	*p*
Lipid metabolism	Serum TC	Low	6	98.7% (< 0.001)	−1.50 (−2.04, −0.95)	< 0.001
		High	2	99.8% (< 0.001)	−3.07 (−8.32, 2.19)	0.253
	Serum TG	Low	6	93.2% (< 0.001)	−0.23 (−0.36, −0.10)	0.001
		High	2	98.4% (< 0.001)	−0.57 (−0.78, 0.08)	0.110
	LDL‐C	Low	4	88.4% (< 0.001)	−0.44 (−0.56, −0.31)	< 0.001
		High	1	NA	−0.26 (−0.43, −0.09)	0.002
	HDL‐C	Low	4	99.6% (< 0.001)	−0.04 (−0.50, 0.41)	0.851
		High	1	NA	0.03 (−0.03, 0.09)	0.338
	Hepatic TC	Low	3	95.9% (< 0.001)	−0.55 (−0.89, −0.21)	0.002
		High	2	88.9% (0.003)	−0.37 (−0.51, −0.23)	< 0.001
	Hepatic TG	Low	4	97.7% (< 0.001)	−0.12 (−0.18, −0, 06)	< 0.001
		High	2	98.0% (< 0.001)	−0.14 (−0.25, −0.03)	0.010
Glucose metabolism	FBG	Low	1	89.9% (< 0.001)	−1.00 (−1.74, −0.25)	0.009
		High	3	NA	−1.04 (−1.34, −0.74)	< 0.001
	INS	Low	3	91.7% (< 0.001)	−5.06 (−9.13, −0.99)	0.015
		High	1	NA	−4.30 (−5.92, −2.69)	< 0.001
Liver enzymes	ALT	Low	6	90.2% (< 0.001)	−2.84 (−4.74, −0.95)	0.003
		High	3	92.6% (< 0.001)	−2.85 (−5.52, −0.18)	0.036
	AST	Low	6	72.0% (0.003)	−4.24 (−5.65, −2.83)	< 0.001
		High	3	93.1% (< 0.001)	−2.60 (−5.28, 0.07)	0.056
Anthropometrics	BW	Low	5	88.7% (< 0.001)	−6.15 (−10.43, −1.87)	0.005
		High	1	NA	−3.80 (−34.13, 26.53)	0.806
	Liver index	Low	4	87.8% (< 0.001)	−0.46 (−0.65, −0.26)	< 0.001
		High	2	95.3% (< 0.001)	−0.99 (−1.93, −0.05)	0.040
Oxidative stress	SOD	Low	2	92.4% (< 0.001)	2.34 (−1.11, 5.79)	0.183
		High	1	NA	17.33 (11.60, 23.05)	< 0.001
	MDA	Low	2	68.9% (0.073)	−2.22 (−3.77, −0.66)	0.005
		High	1	NA	−9.76 (−13.07, −6.46)	< 0.001
Inflammation	TNF‐α	Low	3	86.1% (0.001)	−3.29 (−5.54, −1.04)	0.004
		High	1	NA	−3.21 (−4.58, −1.85)	< 0.001
Signaling molecules	pAMPK/AMPK ratio	Low	2	0.0% (0.692)	4.18 (2.96, 5.40)	< 0.001
		High	1	NA	7.25 (4.79, 9.71)	< 0.001
	SREBP‐1c	Low	2	0.0% (0.600)	−4.37 (−5.62, −3.11)	< 0.001
		High	1	NA	−9.37 (−12.47, −6.27)	< 0.001
	FAS	Low	2	82.5% (0.017)	−3.67 (−6.42, −0.91)	0.009
		High	1	NA	−11.50 (−15.26, −7.74)	< 0.001

Abbreviations: ALT, alanine aminotransferase; AST, aspartate aminotransferase; BW, body weight; FBG, fasting blood glucose; HDL‐C, high‐density lipoprotein cholesterol; INS, insulin; LDL‐C, low‐density lipoprotein cholesterol; MDA, malondialdehyde; SOD, superoxide dismutase; TC, total cholesterol; TG, triglycerides; TNF, tumor necrosis factor.

**TABLE 5:** Subgroup analysis by duration of intervention.

Category	Indicators	Subgroup	Number of experiments	Heterogeneity (*p* value)	SMD/WMD (95% CI)	*p*
Lipid metabolism	Serum TC	Short	7	99.1% (< 0.001)	−2.24 (−2.98, −1.49)	< 0.001
		Long	1	NA	−1.42 (−1.51, −1.33)	< 0.001
	Serum TG	Short	7	93.4% (< 0.001)	−0.29 (−0.42, −0.15)	< 0.001
		Long	1	NA	−0.09 (−0.13, −0.05)	< 0.001
	LDL‐C	Short	4	83.4% (0.001)	−0.45 (−0.63, −0.28)	< 0.001
		Long	1	NA	−0.33 (−0.35, −0.31)	< 0.001
	HDL‐C	Short	4	97.3% (< 0.001)	0.14 (−0.05, 0.32)	0.152
		Long	1	NA	−0.82 (−0.88, −0.76)	< 0.001
	h‐TC	Short	4	98.5% (< 0.001)	−0.29 (−0.48, −0.10)	0.003
		Long	1	NA	−2.07 (−2.68, −1.46)	< 0.001
	h‐TG	Short	5	98.5% (< 0.001)	−0.16 (−0.23, −0.09)	< 0.001
		Long	1	NA	−0.01 (−0.02, 0.01)	0.435
Glucose metabolism	FBG	Short	3	83.4% (0.002)	−0.81 (−1.27, −0.35)	0.001
		Long	1	NA	−1.57 (−2.05, −1.09)	< 0.001
	INS	Short	3	83.4% (0.002)	−3.59 (−5.87, −1.31)	0.002
		Long	1	NA	−8.92 (−11.96, −5.88)	< 0.001
	HOMA‐IR	Short	2	91.2% (0.001)	−3.98 (−8.69, 0.74)	0.098
		Long	1	NA	−10.43 (−13.94, −6.91)	< 0.001
Liver enzymes	ALT	Short	8	90.6% (< 0.001)	−2.79 (−4.34, −1.23)	< 0.001
		Long	1	NA	−3.06 (−4.39, −1.73)	< 0.001
	AST	Short	8	91.2% (< 0.001)	−4.00 (−5.84, −2.15)	< 0.001
		Long	1	NA	−2.83 (−4.11, −1.56)	< 0.001
Anthropometrics	BW	Short	5	88.7% (< 0.001)	−7.95 (−15.75, −0.15)	0.046
		Long	1	NA	−4.36 (−5.22, −3.50)	< 0.001
	Liver index	Short	5	94.0% (< 0.001)	−0.67 (−0.98, −0.37)	< 0.001
		Long	1	NA	−0.41 (−0.55, −0.27)	< 0.001
Signaling molecules	SREBP‐1c	Short	2	88.3% (0.003)	−6.54 (−11.77, −1.32)	0.014
		Long	1	NA	−4.70 (−6.47, −2.93)	< 0.001
	FAS	Short	2	95.1% (< 0.001)	−6.75 (−15.71, 2.21)	0.140
		Long	1	NA	−5.16 (−7.07, −3.26)	< 0.001

Abbreviations: ALT, alanine aminotransferase; AST, aspartate aminotransferase; BW, body weight; FBG, fasting blood glucose; HDL‐C, high‐density lipoprotein cholesterol; INS, insulin; LDL‐C, low‐density lipoprotein cholesterol; MDA, malondialdehyde; SOD, superoxide dismutase; TC, total cholesterol; TG, triglycerides.

**TABLE 6:** Subgroup analysis by route of administration.

Category	Indicators	Subgroup	Number of experiments	Heterogeneity (*p* value)	SMD/WMD (95% CI)	*p*
Lipid metabolism	Serum TC	Gavage	7	99.1% (< 0.001)	−2.24 (−2.98, −1.49)	< 0.001
		Dietary	1	NA	−1.42 (−1.51, −1.33)	< 0.001
	Serum TG	Gavage	7	93.4% (< 0.001)	−0.29 (−0.42, −0.15)	< 0.001
		Dietary	1	NA	−0.09 (−0.13, −0.05)	< 0.001
	LDL‐C	Gavage	4	83.4% (< 0.001)	−0.45 (−0.63, −0.28)	< 0.001
		Dietary	1	NA	−0.33 (−0.35, −0.31)	< 0.001
	HDL‐C	Gavage	4	97.3% (< 0.001)	0.14 (−0.05, 0.32)	0.152
		Dietary	1	NA	−0.82 (−0.88, −0.76)	< 0.001
	h‐TC	Gavage	4	98.5% (< 0.001)	−0.29 (−0.48, −0.10)	0.003
		Dietary	1	NA	−2.07 (−2.68, −1.46)	< 0.001
	h‐TG	Gavage	5	98.5% (< 0.001)	−0.16 (−0.23, −0.09)	< 0.001
		Dietary	1	NA	−0.01 (−0.02, 0.01)	0.435
Glucose metabolism	FBG	Gavage	3	83.4% (0.002)	−0.81 (−1.27, −0.35)	0.001
		Dietary	1	NA	−1.57 (−2.05, −1.09)	< 0.001
	INS	Gavage	3	83.4% (0.002)	−3.59 (−5.87, −1.31)	0.002
		Dietary	1	NA	−8.92 (−11.96, −5.88)	< 0.001
	HOMA‐IR	Gavage	2	91.2% (0.001)	−3.98 (−8.69, 0.74)	0.098
		Dietary	1	NA	−10.43 (−13.94, −6.91)	< 0.001
Liver enzymes	ALT	Gavage	8	90.6% (< 0.001)	−2.79 (−4.34, −1.23)	< 0.001
		Dietary	1	NA	−3.06 (−4.39, −1.73)	< 0.001
	AST	Gavage	8	91.2% (< 0.001)	−4.00 (−5.84, −2.15)	< 0.001
		Dietary	1	NA	−2.83 (−4.11, −1.56)	< 0.001
Anthropometrics	BW	Gavage	5	88.7% (< 0.001)	−7.95 (−15.75, −0.15)	0.046
		Dietary	1	NA	−4.36 (−5.22, −3.50)	< 0.001
	Liver index	Gavage	5	94.0% (< 0.001)	−0.67 (−0.98, −0.37)	< 0.001
		Dietary	1	NA	−0.41 (−0.55, −0.27)	< 0.001
Signaling molecules	SREBP‐1c	Gavage	2	88.3% (0.003)	−6.54 (−11.77, −1.32)	0.014
		Dietary	1	NA	−4.70 (−6.47, −2.93)	< 0.001
	FAS	Gavage	2	95.1% (< 0.001)	−6.75 (−15.71, 2.21)	0.140
		Dietary	1	NA	−5.16 (−7.07, −3.26)	< 0.001

Abbreviations: ALT, alanine aminotransferase; AST, aspartate aminotransferase; BW, body weight; FBG, fasting blood glucose; HDL‐C, high‐density lipoprotein cholesterol; INS, insulin; LDL‐C, low‐density lipoprotein cholesterol; MDA, malondialdehyde; SOD, superoxide dismutase; TC, total cholesterol; TG, triglycerides.

However, despite these factors mitigating some heterogeneity, other unaccounted variables, such as differences in animal age, sex, experimental environment, disease model establishment, and data quality reporting, may still contribute to observed variability. Addressing these factors in future studies could help minimize heterogeneity and provide more consistent findings in preclinical research on Paeoniflorin for NAFLD.

### 3.6 Sensitivity Analysis

Sensitivity analyses were conducted for outcomes for which data from at least five studies were available, including lipid metabolism markers, liver enzymes, and anthropometric markers (Figure S4–S6) (Chandler et al. 2019). In each analysis, the meta‐analysis was performed after sequentially excluding one study at a time. This approach allowed us to assess the stability and robustness of the pooled results. The findings from the sensitivity analyses indicated that the overall effect of Paeoniflorin supplementation on these markers remained consistent across all iterations. Specifically, excluding individual studies did not significantly alter the overall effect sizes, reinforcing the reliability of the results. Sensitivity analyses were not performed for inflammatory and oxidative stress markers or signaling molecules because fewer than five studies were available for these outcomes. Since fewer than 10 studies were available for each outcome, the prespecified publication bias analysis was not performed. According to the Cochrane Handbook for Systematic Reviews of Interventions, such tests are considered unreliable with smaller sample sizes and could lead to misleading conclusions.

Additional lowest‐dose sensitivity analyses were performed for outcomes in which at least one included study had multiple eligible PF dose arms. Compared with the primary analyses based on the highest‐dose arm, the lowest‐dose analyses showed directions of effect consistent with the primary analyses for all evaluated outcomes. Some pooled effects remained statistically significant, whereas others showed attenuation of effect size or loss of statistical significance. These findings suggest that the main conclusions were not driven solely by preferential extraction of the highest PF dose, although the pooled estimates should still be interpreted as directional preclinical evidence rather than precise dose‐independent effect sizes. Forest plots for these lowest‐dose sensitivity analyses are presented in Figures S7–S12.

Galbraith plot analyses were further conducted for outcomes with at least five studies. Several highly heterogeneous outcomes showed one or more studies lying outside the expected limits, indicating that between‐study variability was partly driven by individual study‐level differences. These findings were consistent with the substantial heterogeneity observed in the primary analyses and supported cautious interpretation of pooled effect sizes. The Galbraith plots are presented in Figures S13–S15.

Prediction interval analyses were additionally performed for the pooled outcomes. For several highly heterogeneous outcomes, prediction intervals were substantially wider than the corresponding 95% confidence intervals, indicating that the magnitude of treatment effects may vary across future experimental settings. Nevertheless, the direction of effect generally remained consistent with the primary pooled estimates.

## 4 Discussion

### 4.1 Overview of Principal Findings

Our systematic review and meta‐analysis of preclinical studies on paeoniflorin (PF) supplementation in animal models of non‐alcoholic fatty liver disease (NAFLD) revealed promising therapeutic effects. PF treatment was associated with improvements in multiple NAFLD‐related outcomes, including liver enzyme reduction (ALT and AST), lipid metabolism regulation (TC, TG, and LDL‐C), and enhanced glucose metabolism (FBG, insulin and HOMA‐IR). Furthermore, PF was also associated with anti‐inflammatory and antioxidant effects, including lowering TNF‐α and MDA levels and higher SOD activity. PF also reduced body weight and liver index, whereas HDL‐C did not show a significant change. The findings were marked by considerable heterogeneity, suggesting that species, dosing regimen, treatment duration, and administration route may influence the magnitude of observed effects.

### 4.2 Possible Mechanisms of PF's Protective Effects

#### 4.2.1 Regulation of Lipid Metabolism and AMPK Activation

One important finding of this review was the consistent association between PF and improvements in lipid metabolism. Our results suggested reductions in circulating and hepatic lipid burdens, which are accompanied by improvements in liver injury markers, suggesting that PF alleviates steatosis‐related metabolic stress and downstream hepatocellular damage. Consistent with this lipid‐improving phenotype, evidence synthesized in our review indicates that PF is associated with enhanced AMPK signaling, reflected by increased phosphorylation of AMPK. Mechanistically, AMPK is widely recognized as a master regulator of cellular energy homeostasis and an attractive target for metabolic disease therapy (Townsend and Steinberg 2023). Once activated, AMPK can repress de novo lipogenesis partly by inhibiting SREBP‐1c, a transcription factor that drives the expression of enzymes involved in fatty acid and cholesterol synthesis (Wang et al. 2025). In parallel, AMPK activation can reduce the activity and/or expression of downstream lipogenic enzymes such as FAS (Wu et al. 2018), providing a coherent explanation for PF‐associated reductions in lipid accumulation. Beyond restraining lipogenesis, AMPK also promotes fatty‐acid utilization. It can enhance pathways that facilitate mitochondrial β‐oxidation, including regulation of CPT‐1‐dependent fatty‐acid entry into mitochondria (Deja et al. 2024). This shift toward lipid oxidation may further reduce hepatic steatosis and improve systemic lipid profiles, complementing PF's lipid‐lowering effects observed across preclinical studies. However, these signaling outcomes were reported in only a small number of studies and should be interpreted as supportive mechanistic signals rather than definitive evidence of pathway‐specific effects.

#### 4.2.2 Anti‐Inflammatory and Antioxidant Effects

Chronic inflammation and oxidative stress are central to the progression of NAFLD (Li et al. 2016). In this meta‐analysis, PF was associated with lower TNF‐α and MDA levels and higher SOD activity, suggesting anti‐inflammatory and antioxidative signals in animal models. These findings are consistent with individual studies reporting that PF may modulate NF‐κB/NLRP3‐related inflammatory pathways and antioxidant responses. For example, Ma et al. (2016) reported that PF reduced oxidative stress and inflammation in a NASH model through inhibition of NF‐κB activation and enhancement of SOD activity.

These findings should be interpreted as exploratory rather than definitive mechanistic evidence. TNF‐α, oxidative stress, and mitochondrial dysfunction are biologically linked in NAFLD, and PF‐related changes in these markers may reflect attenuation of the inflammatory–oxidative stress loop (Li et al. 2016; Vachliotis and Polyzos 2023; Clare et al. 2022; Karkucinska‐Wieckowska et al. 2022). However, the number of studies measuring these endpoints was small, and related pathways were not assessed uniformly across experiments. Therefore, the current evidence supports a possible anti‐inflammatory and antioxidative role of PF, but does not establish a single causal pathway.

PF's anti‐inflammatory activity may not be limited to hepatic cytokine changes. In LPS‐stimulated splenocytes and splenic CD4+ T lymphocytes from MRL/lpr mice, PF suppressed IRAK1–NF‐κB signaling and reduced downstream inflammatory cytokine expression, with a more evident effect in CD4+ T lymphocytes (Ji et al. 2022). Another study in dimethylnitrosamine‐induced liver fibrosis showed that PF affected macrophage activation, as indicated by changes in CD68 expression in the liver as well as in extrahepatic organs, including the spleen and lung (Chen et al. 2012). Although these models are not NAFLD‐specific, they are relevant to the inflammatory background of metabolic liver disease. NAFLD has been discussed in the context of a liver–spleen axis, in which spleen‐related immune regulation may contribute to chronic low‐grade inflammation and insulin resistance (Tarantino et al. 2021). These observations suggest that the anti‐inflammatory signal observed in PF‐treated NAFLD models may involve not only local hepatic pathways, but also immune‐cell regulation across liver–spleen crosstalk. This interpretation remains indirect, however, and should be tested in dedicated NAFLD models.

#### 4.2.3 Effects on Glucose Metabolism and Insulin Sensitivity

PF may improve glucose metabolism and insulin sensitivity through coordinated effects on AMPK‐ and Akt‐related signaling. In NAFLD models, PF has been linked to activation of the LKB1/AMPK axis and involvement of Akt signaling (Li et al. 2018; Wen et al. 2019). AMPK activation may reduce hepatic insulin resistance by promoting oxidative metabolism and limiting lipotoxic intermediates that interfere with insulin signaling (Fullerton et al. 2013). In addition, one NAFLD study reported modulation of the IRS/Akt/GSK3β pathway after PF treatment (Ma et al. 2017). Together with reduced inflammatory and oxidative stress signals, these changes suggest a possible insulin‐sensitizing effect of PF, although the evidence remains limited and does not establish a single causal pathway.

#### 4.2.4 Lipid Peroxidation and Mitochondrial Function

As a terminal product of lipid peroxidation, MDA reflects oxidative damage to polyunsaturated fatty acids and provides a mechanistically relevant readout of membrane injury in NAFLD (Yang et al. 2019). In our meta‐analysis, PF significantly reduced MDA, suggesting attenuation of lipid peroxidation within hepatic tissues. This is important because lipid peroxidation products can disrupt mitochondrial membrane integrity, compromise respiratory chain function, and decrease oxidative phosphorylation efficiency, thereby aggravating hepatocellular stress and injury (Gupta et al. 2017). Consistent with a mitochondria‐centered interpretation, experimental studies have reported that PF improves mitochondrial homeostasis in hepatocytes, including normalization of mitochondrial dynamics (fusion–fission balance) and enhancement of oxidative phosphorylation capacity (Li et al. 2022; Fei et al. 2025). By limiting lipid peroxidation and preserving mitochondrial performance, PF may help interrupt the feed‐forward deterioration in which membrane damage and mitochondrial impairment amplify metabolic stress and liver injury in NAFLD.

#### 4.2.5 Hepatocellular Injury and Organ‐Level Phenotypes

ALT and AST reflect hepatocellular injury rather than disease‐specific mechanisms. Their reduction in this meta‐analysis is biologically consistent with the observed improvements in hepatic lipid burden, lipogenic signaling, inflammation, and oxidative stress but should still be interpreted cautiously because enzyme responses may vary with model severity, assay platforms, and intervention timing (Metra et al. 2022; Karatas et al. 2023; Perla et al. 2017; Liu et al. 2016; Kudo et al. 2022; Dawson et al. 2023; Yamasaki et al. 2023; Turkseven et al. 2023; Fang et al. 2023; Atteia et al. 2023). Importantly, aminotransferase improvements do not necessarily indicate histological resolution, underscoring the need for harmonized NAS and fibrosis endpoints (Ratziu et al. 2021; Ji et al. 2021; Loomba et al. 2024).

The concurrent reductions in body weight and liver index suggest that PF may influence both systemic and hepatic phenotypes in NAFLD animal models. Liver index can reflect hepatic lipid deposition, hepatocyte hypertrophy or ballooning, inflammatory infiltration, and edema, whereas body weight is affected by diet composition, baseline obesity, food intake, treatment duration, and body composition (Zhao et al. 2022; Zhang, Yu, et al. 2024; Axelrod et al. 2023; Kuang et al. 2023; Wu et al. 2022). Because these factors were incompletely reported across studies, the body‐weight and liver‐index findings should be interpreted cautiously. Future studies should report energy intake, adiposity or body composition, and standardized histological scores to clarify whether these changes correspond to durable improvement in steatohepatitis or early fibrotic remodeling.

### 4.3 Limitations and Future Directions

Although this review was based on a preregistered protocol and conducted in accordance with PRISMA 2020, several considerations warrant cautious interpretation. First, the number of included studies was limited and individual studies generally had small sample sizes, which constrains the precision of pooled estimates for some outcomes. Second, substantial between‐study heterogeneity was observed for many outcomes, which is common in animal research and suggests that effect sizes may vary with species/model characteristics, dosing regimen and duration, route of administration, and outcome assessment methods. We performed prespecified subgroup analyses to explore potential sources of heterogeneity; however, given the limited number of studies, these findings should be viewed as exploratory and hypothesis‐generating. Third, multiple key domains were judged as “unclear risk” in the SYRCLE assessment, largely because methodological details such as randomization, allocation concealment, and blinding were insufficiently reported rather than because of documented methodological deficiencies. Future studies should improve reporting transparency to enhance reproducibility and facilitate more reliable assessment of study quality. Consequently, the pooled estimates reported here should be interpreted with caution, as inadequate reporting of these safeguards may have contributed to overestimation of treatment effects. Fourth, as prespecified, formal tests for publication bias were not performed for most outcomes because fewer than 10 studies were available, and such tests are considered unreliable with small numbers of studies. Fifth, geographical concentration is another limitation: all included studies were conducted in China, which may limit external validity and reproducibility across different laboratory settings and research groups. Replication in independent cohorts and broader research contexts is needed to strengthen confidence in the generalizability of these preclinical signals. Finally, to avoid unit‐of‐analysis errors arising from shared control groups, we extracted data from the highest‐dose arm when multiple dose arms were reported within the same study. Although this approach preserves statistical independence, it may preferentially reflect near‐maximal effects. To address this concern, we added lowest‐dose sensitivity analyses where alternative PF dose arms were available. The main direction of effect was preserved across all evaluated outcomes, although attenuation of some estimates or loss of statistical significance indicates that the pooled effects should not be interpreted as dose‐independent treatment effects. In addition, Galbraith plot analyses suggested that selected highly heterogeneous outcomes were influenced by individual study‐level differences. These diagnostic analyses reinforce the need to interpret the results as directional preclinical evidence rather than precise estimates of efficacy. Future preclinical studies should include adequately powered multi‐dose designs, standardized histological endpoints, and more complete reporting of outcomes to permit formal dose–response modeling and stage‐specific evidence synthesis. In addition, prediction intervals were wide for several outcomes, indicating that the magnitude of treatment effects may vary substantially across future experimental settings despite a generally consistent direction of effect.

## 5 Conclusions

Overall, pooled preclinical evidence suggests that paeoniflorin (PF) is associated with improvements in hepatic lipid accumulation (with accompanying AMPK‐related signaling changes), body weight, liver index, liver injury enzymes, insulin resistance indices, inflammatory signaling (TNF‐α), and oxidative stress markers (MDA and SOD) in NAFLD models. Species, dose, duration, and administration route may partly explain variability in effects. Standardized, rigorously reported animal studies and well‐designed clinical investigations are needed to determine whether these benefits translate to human NAFLD.

## Author Contributions

**Guangming Li:** project administration, validation. **Tao Zhou:** data curation, investigation. **Peng Gao:** methodology, software. **Shunqin Jin:** data curation, investigation. **Chuan Qin:** validation, project administration, supervision. **Mingfei Yao:** methodology, software. **Dachuan Jin:** writing – original draft, writing – review and editing, formal analysis. **Guoping Sheng:** data curation, investigation, conceptualization.

## Funding

This work was supported by the National Key Research and Development Program (2023ZD0502400, 2024YFA1307100), the Natural Science Foundation of Shandong Province (Grant No. ZR2023MH147), and the 2025 Zhengzhou Municipal Science and Technology Innovation Guidance Program Project in the Medical and Health Field (Grant No. 2025YLZDJH110). The funders had no role in study design, data collection and analysis, decision to publish, or preparation of the manuscript.

## Ethics Statement

The authors have nothing to report.

## Conflicts of Interest

The authors declare no conflicts of interest.

## Supporting information


**Figure S1:** PRISMA 2020 flow diagram of the study selection process.
**Figure S2:** Risk‐of‐bias graph for the included animal studies. Proportions of studies rated as having low, unclear, or high risk of bias across each domain using SYRCLE's risk‐of‐bias tool.
**Figure S3:** Risk‐of‐bias summary for the included animal studies. The traffic‐light plot shows the risk‐of‐bias judgment for each SYRCLE domain in each included study.
**Figure S4:** Sensitivity analysis of lipid metabolism markers. Leave‐one‐out sensitivity analyses were performed to evaluate the robustness of the pooled estimates; the summary effect was recalculated after sequentially omitting one study at a time. HDL‐C, high‐density lipoprotein cholesterol; h‐TC, hepatic total cholesterol; h‐TG, hepatic total triglyceride; LDL‐C, low‐density lipoprotein cholesterol; s‐TC, serum total cholesterol; s‐TG, serum triglyceride.
**Figure S5:** Sensitivity analysis of liver enzymes. Leave‐one‐out sensitivity analyses were performed to evaluate the robustness of the pooled estimates; the summary effect was recalculated after sequentially omitting one study at a time. ALT, alanine aminotransferase. AST, aspartate aminotransferase.
**Figure S6:** Sensitivity analysis of anthropometric outcomes. Leave‐one‐out sensitivity analyses were performed to evaluate the robustness of the pooled estimates; the summary effect was recalculated after sequentially omitting one study at a time. BW, body weight.
**Figure S7:** Forest plots of the lowest‐dose sensitivity analyses for lipid metabolism outcomes. HDL‐C, high‐density lipoprotein cholesterol; LDL‐C, low‐density lipoprotein cholesterol; TC, total cholesterol; TG, triglycerides; WMD, weighted mean differences.
**Figure S8:** Forest plots of the lowest‐dose sensitivity analyses for liver enzyme outcomes. ALT, alanine aminotransferase; AST, aspartate aminotransferase.
**Figure S9:** Forest plots of the lowest‐dose sensitivity analyses for anthropometric outcomes.
**Figure S10:** Forest plots of the lowest‐dose sensitivity analyses for glucose metabolism outcomes. FBG, fasting blood glucose; HOMA‐IR, homeostatic model assessment of insulin resistance.
**Figure S11:** Forest plots of the lowest‐dose sensitivity analyses for inflammation and oxidative stress markers. MDA, malondialdehyde; TNF‐α, tumor necrosis factor‐α; SOD, superoxide dismutase.
**Figure S12:** Forest plots of the lowest‐dose sensitivity analyses for signaling molecules. FAS, fatty acid synthase; pAMPK/AMPK, phosphorylated AMP‐activated protein kinase/AMP‐activated protein kinase ratio; SREBP‐1c, sterol regulatory element‐binding protein‐1c.
**Figure S13:** Galbraith plots for lipid metabolism outcomes. HDL‐C, high‐density lipoprotein cholesterol; LDL‐C, low‐density lipoprotein cholesterol; TC, total cholesterol; TG, triglycerides.
**Figure S14:** Galbraith plots for liver enzyme outcomes. ALT, alanine aminotransferase; AST, aspartate aminotransferase.
**Figure S15:** Galbraith plots for anthropometric outcomes. BW, body weight.

## Data Availability

The analytical dataset supporting the meta‐analysis, together with the corresponding subgroup‐analysis forest plots and figure legends, is publicly available on Figshare at https://doi.org/10.6084/m9.figshare.33028130.
