# Supplementary material for: Paeoniflorin and NAFLD: A Systematic Review and Meta‐Analysis of Animal Studies With Mechanistic Insights
Source: Food Sci Nutr. 2026 Aug 3;14(8):e72192. doi: 10.1002/fsn3.72192 (PMC13430278; doi:10.1002/fsn3.72192)
Supplement: Supplementary file 1 — Figure S1: PRISMA 2020 flow diagram of the study selection process. Figure S2: Risk‐of‐bias graph for the included animal studies. Proportions of studies rated as having low, unclear, or high risk of bias across each domain using SYRCLE's risk‐of‐bias tool. Figure S3: Risk‐of‐bias summary for the included animal studies. The traffic‐light plot shows the risk‐of‐bias judgment for each SYRCLE domain in each included study. Figure S4: Sensitivity analysis of lipid metabolism markers. Leave‐one‐out sensitivity analyses were performed to evaluate the robustness of the pooled estimates; the summary effect was recalculated after sequentially omitting one study at a time. HDL‐C, high‐density lipoprotein cholesterol; h‐TC, hepatic total cholesterol; h‐TG, hepatic total triglyceride; LDL‐C, low‐density lipoprotein cholesterol; s‐TC, serum total cholesterol; s‐TG, serum triglyceride. Figure S5: Sensitivity analysis of liver enzymes. Leave‐one‐out sensitivity analyses were performed to evaluate the robustness of the pooled estimates; the summary effect was recalculated after sequentially omitting one study at a time. ALT, alanine aminotransferase. AST, aspartate aminotransferase. Figure S6: Sensitivity analysis of anthropometric outcomes. Leave‐one‐out sensitivity analyses were performed to evaluate the robustness of the pooled estimates; the summary effect was recalculated after sequentially omitting one study at a time. BW, body weight. Figure S7: Forest plots of the lowest‐dose sensitivity analyses for lipid metabolism outcomes. HDL‐C, high‐density lipoprotein cholesterol; LDL‐C, low‐density lipoprotein cholesterol; TC, total cholesterol; TG, triglycerides; WMD, weighted mean differences. Figure S8: Forest plots of the lowest‐dose sensitivity analyses for liver enzyme outcomes. ALT, alanine aminotransferase; AST, aspartate aminotransferase. Figure S9: Forest plots of the lowest‐dose sensitivity analyses for anthropometric outcomes. Figure S10: Forest plots of the lowest‐dose [file FSN3-14-e72192-s001.docx]

***Supplementary Material***

**
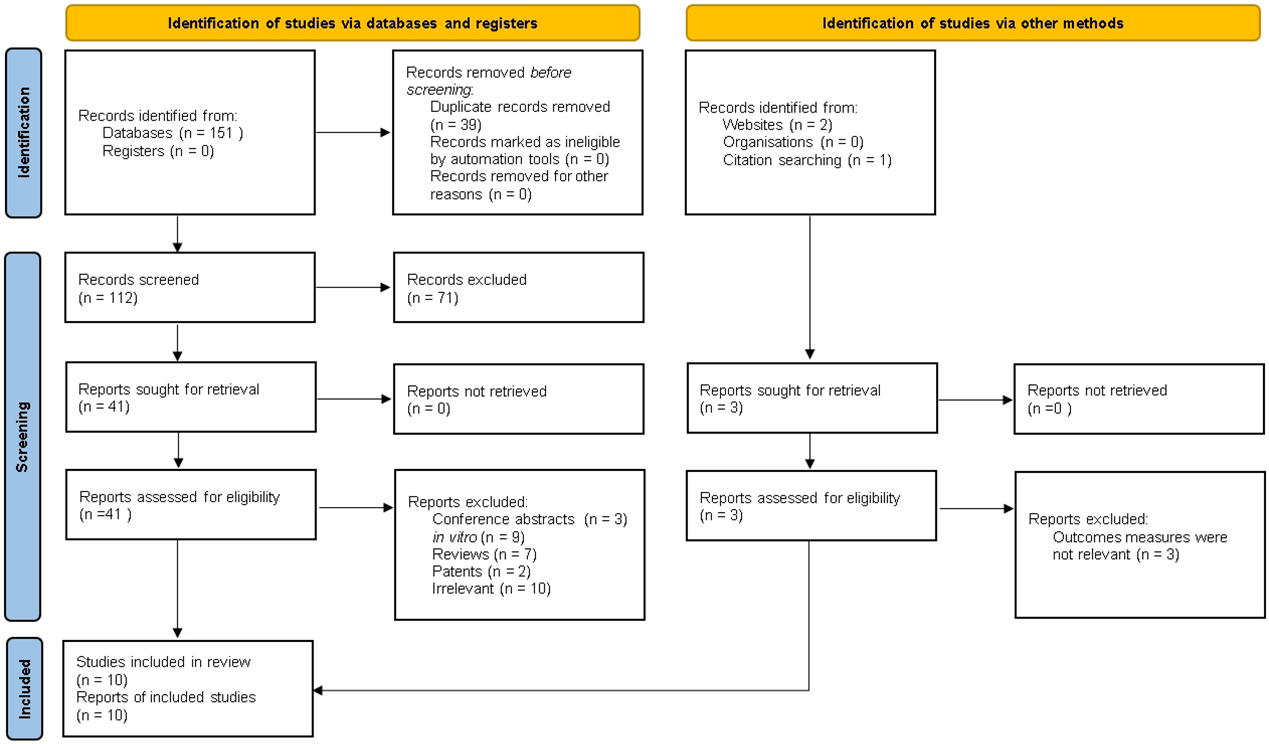
**

**Figure S1 PRISMA 2020 flow diagram of the study selection process.**

**
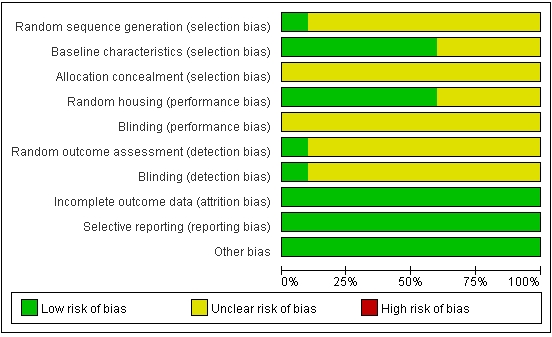
**

**Figure S2 Risk-of-bias graph for the included animal studies.** Proportions of studies rated as having low, unclear, or high risk of bias across each domain using SYRCLE’s risk-of-bias tool.

**
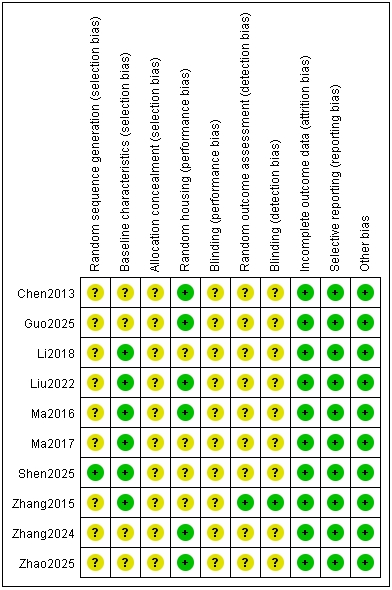
**

**Figure S3 Risk-of-bias summary for the included animal studies.** The traffic-light plot shows the risk-of-bias judgment for each SYRCLE domain in each included study.

**
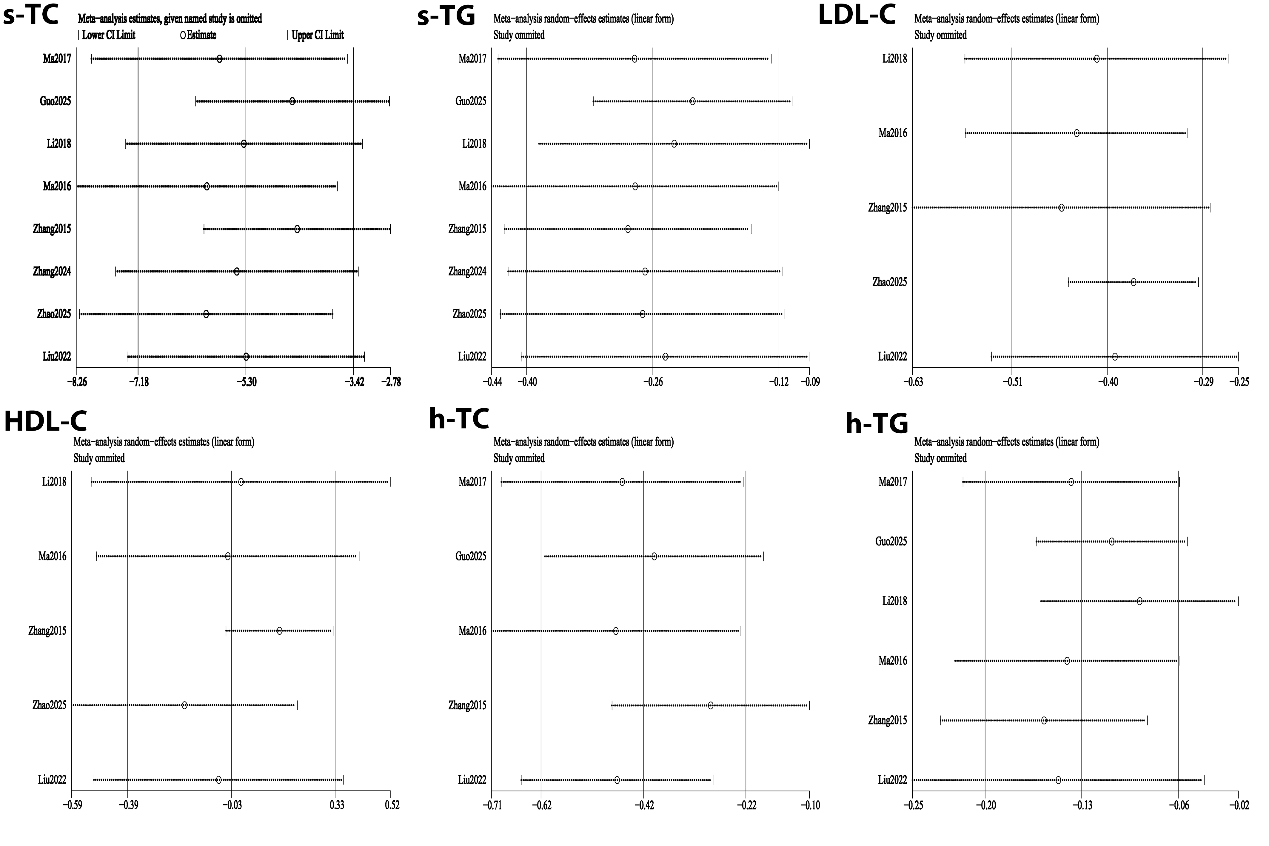
**

**Figure S4 Sensitivity analysis of lipid metabolism markers.** Leave-one-out sensitivity analyses were performed to evaluate the robustness of the pooled estimates; the summary effect was recalculated after sequentially omitting one study at a time. s-TC, serum total cholesterol; s-TG, serum triglyceride; LDL-C, low-density lipoprotein cholesterol; HDL-C, high-density lipoprotein cholesterol; h-TC, hepatic total cholesterol; h-TG, hepatic total triglyceride.

**
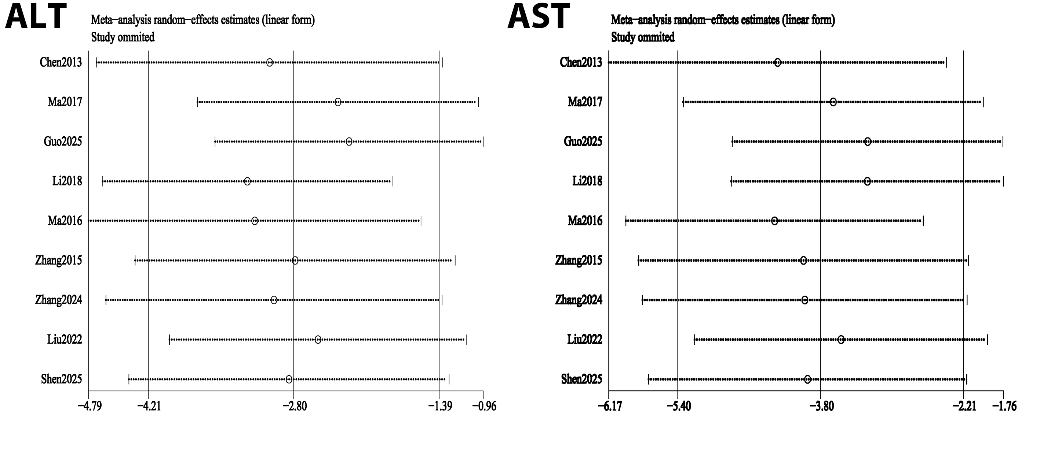
**

**Figure S5 Sensitivity analysis of liver enzymes.** Leave-one-out sensitivity analyses were performed to evaluate the robustness of the pooled estimates; the summary effect was recalculated after sequentially omitting one study at a time. ALT, alanine aminotransferase. AST, aspartate aminotransferase.

**
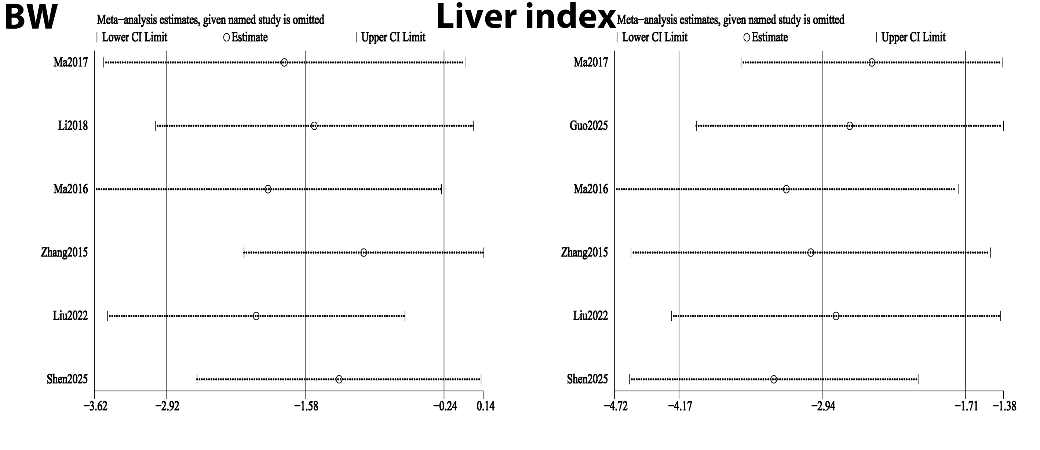
**

**Figure S6 Sensitivity analysis of anthropometric outcomes.** Leave-one-out sensitivity analyses were performed to evaluate the robustness of the pooled estimates; the summary effect was recalculated after sequentially omitting one study at a time. BW, body weight.


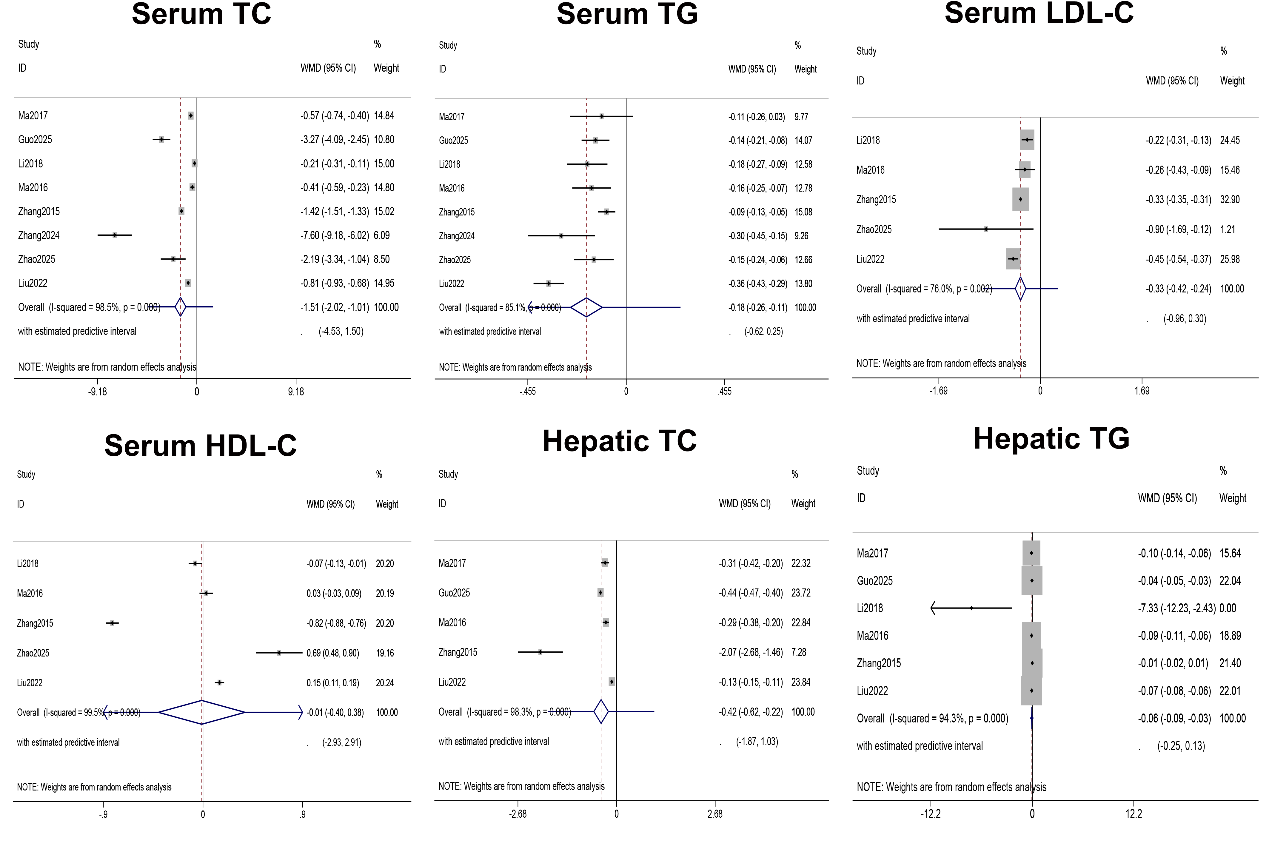


**Figure S7. Forest plots of the lowest-dose sensitivity analyses for lipid metabolism outcomes.** WMD, weighted mean differences; TC, total cholesterol; TG, triglycerides; LDL-C, low-density lipoprotein cholesterol; HDL-C, high-density lipoprotein cholesterol.


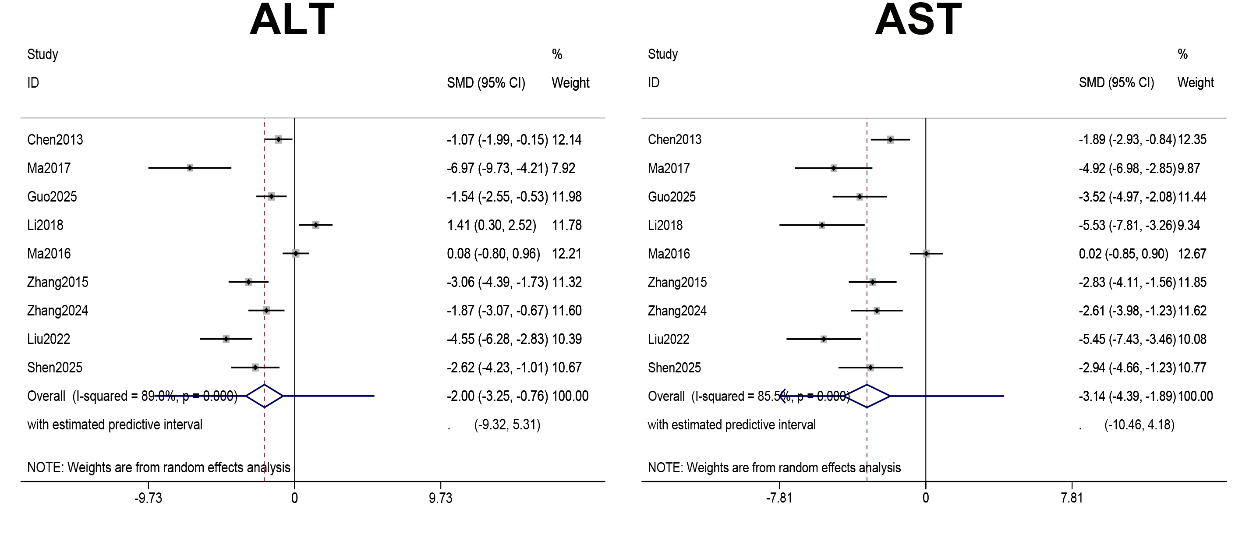
 **Figure S8. Forest plots of the lowest-dose sensitivity analyses for liver enzyme outcomes.** ALT, alanine aminotransferase; AST, aspartate aminotransferase.

**
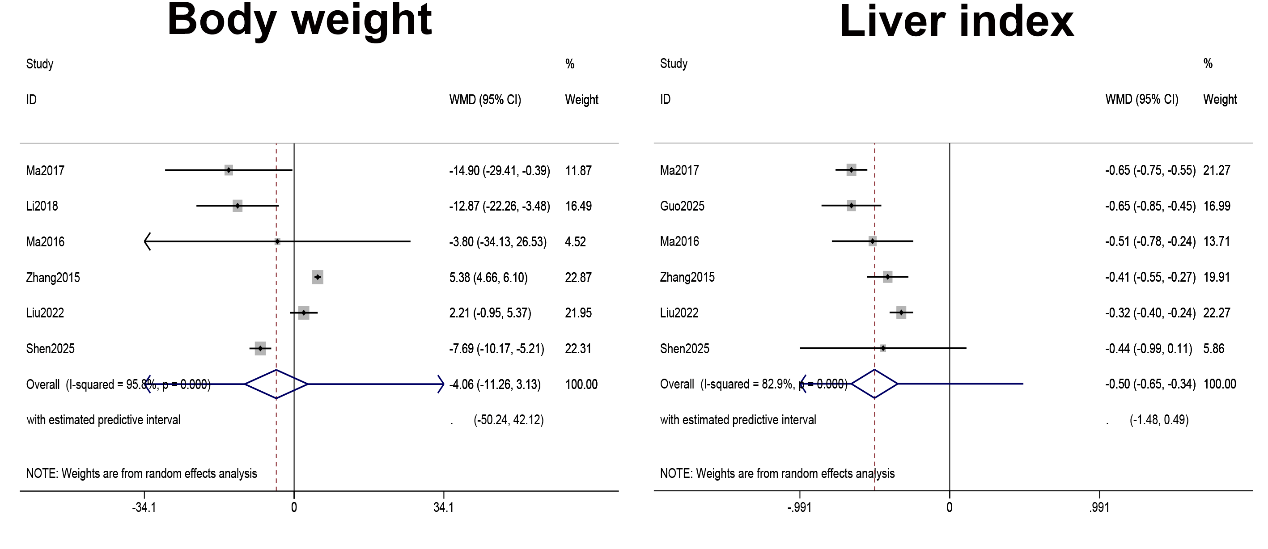
**

**Figure S9. Forest plots of the lowest-dose sensitivity analyses for anthropometric outcomes.**

**
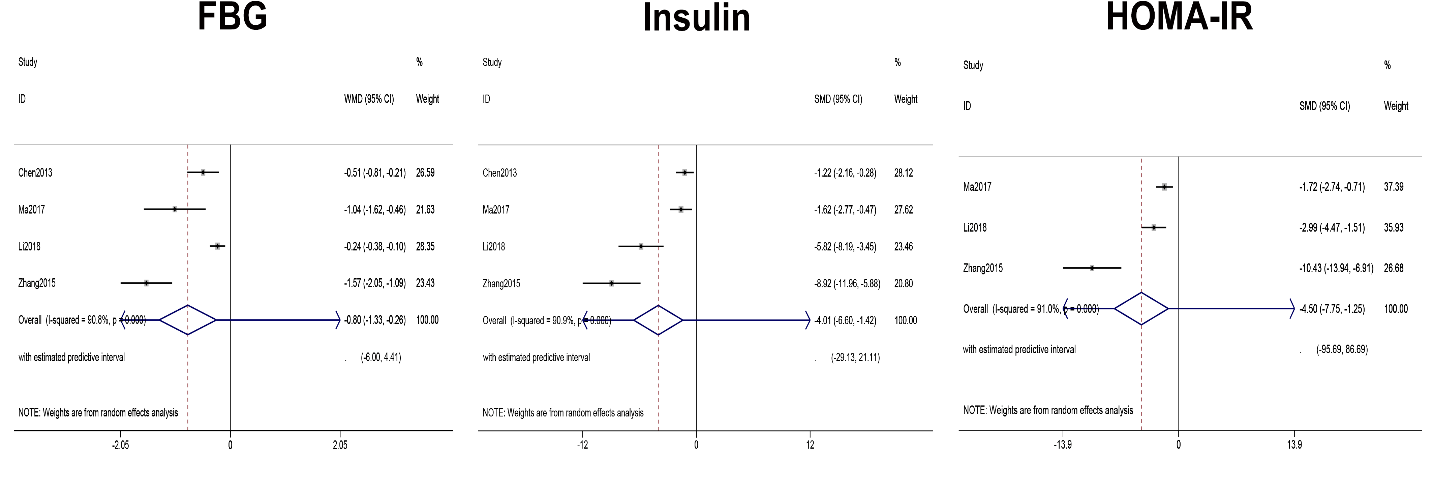
**

**Figure S10. Forest plots of the lowest-dose sensitivity analyses for glucose metabolism outcomes.** FBG, fasting blood glucose; HOMA-IR, homeostatic model assessment of insulin resistance.

**
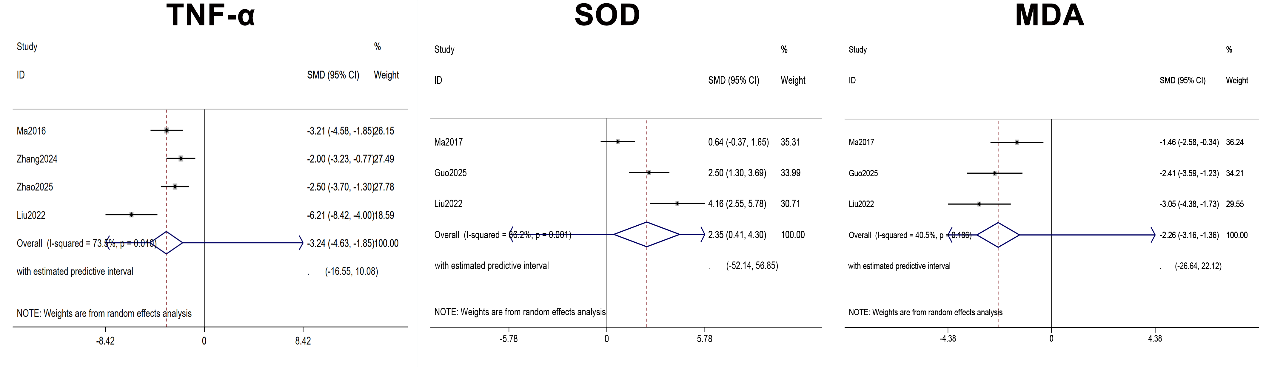
**

**Figure S11. Forest plots of the lowest-dose sensitivity analyses for inflammation and oxidative stress markers.** TNF-α, tumor necrosis factor-α; SOD, superoxide dismutase; MDA, malondialdehyde.

**
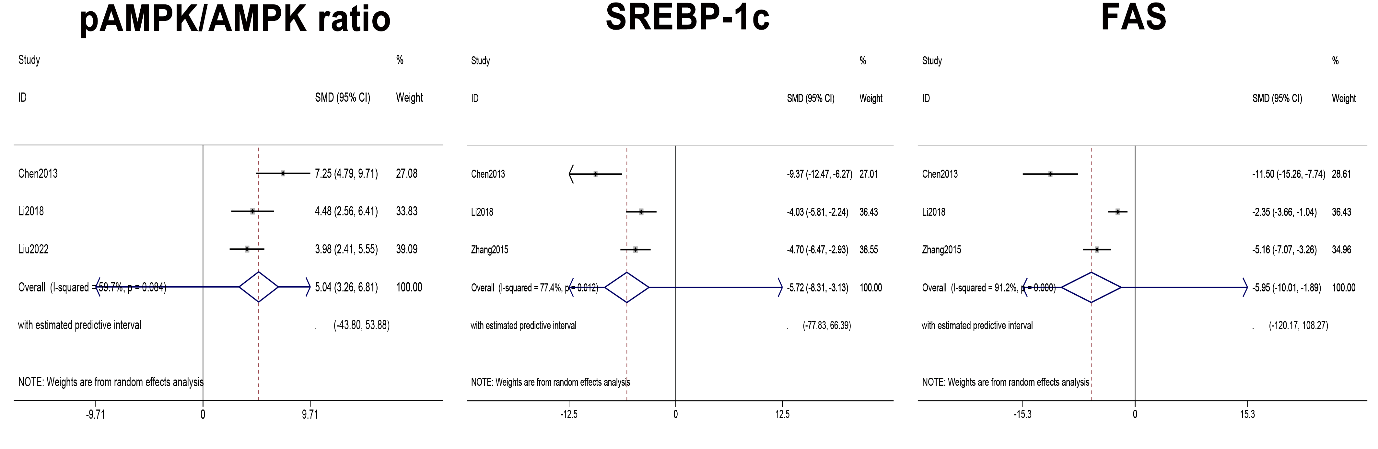
** **Figure S12. Forest plots of the lowest-dose sensitivity analyses for signaling molecules.** pAMPK/AMPK, phosphorylated AMP-activated protein kinase/AMP-activated protein kinase ratio; SREBP-1c, sterol regulatory element-binding protein-1c; FAS, fatty acid synthase.


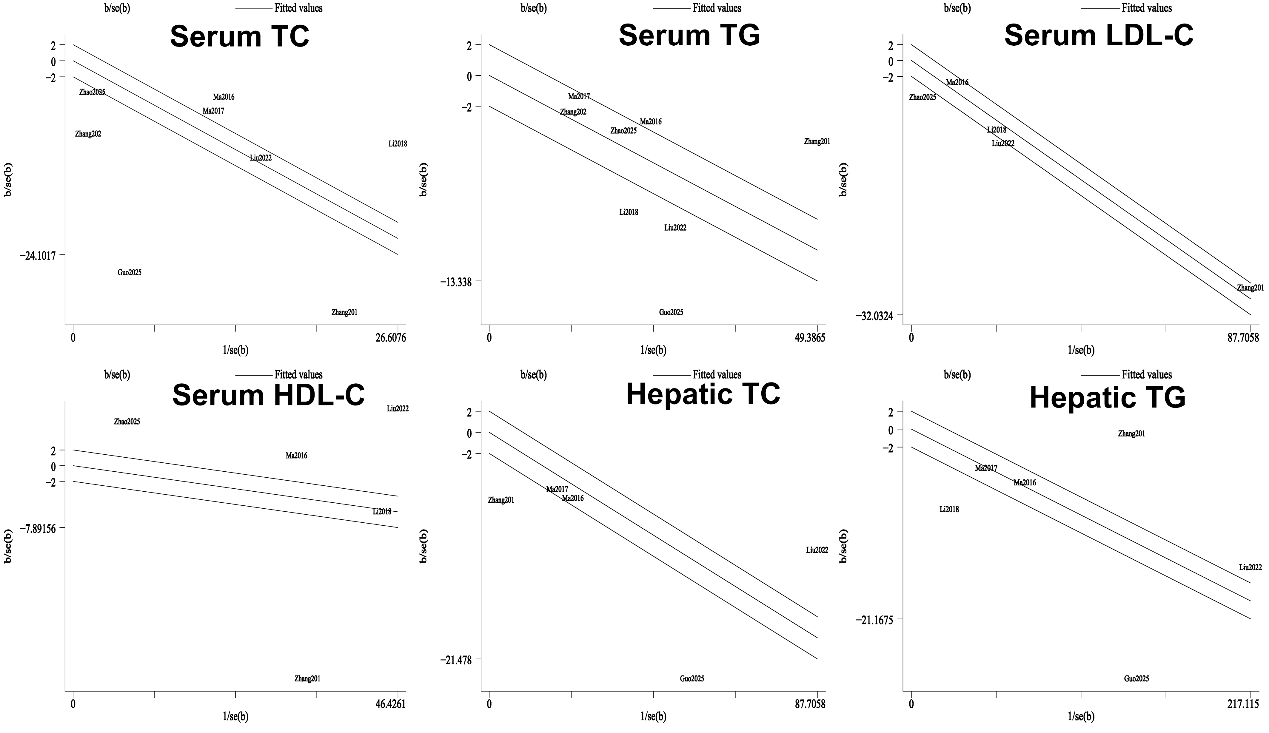


**Figure S13. Galbraith plots for lipid metabolism outcomes.** TC, total cholesterol; TG, triglycerides; LDL-C, low-density lipoprotein cholesterol; HDL-C, high-density lipoprotein cholesterol.


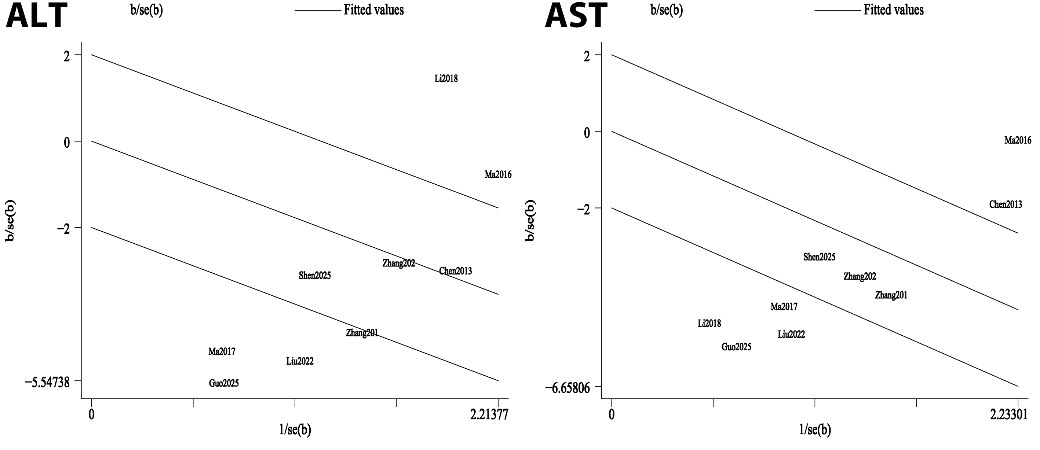
 **Figure S14. Galbraith plots for liver enzyme outcomes.** ALT, alanine aminotransferase; AST, aspartate aminotransferase.


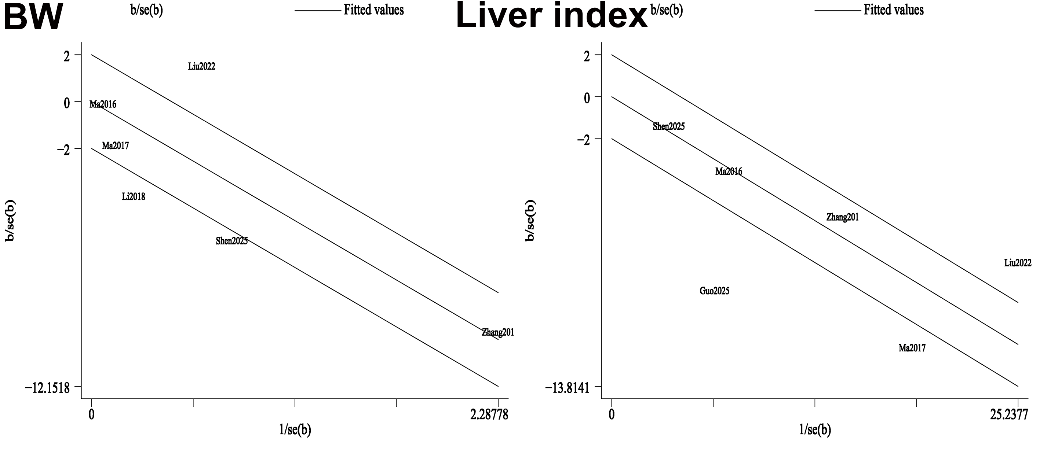


**Figure S15. Galbraith plots for anthropometric outcomes.** BW, body weight.
